# Elucidating the Mechanism of Fe Incorporation in In Situ Synthesized Co–Fe Oxygen-Evolving Nanocatalysts

**DOI:** 10.1021/jacs.3c08099

**Published:** 2023-10-20

**Authors:** Thi Ha My Pham, Tzu-Hsien Shen, Youngdon Ko, Liping Zhong, Loris Lombardo, Wen Luo, Satoshi Horike, Vasiliki Tileli, Andreas Züttel

**Affiliations:** † Laboratory of Materials for Renewable Energy (LMER), Institute of Chemical Sciences and Engineering (ISIC), Basic Science Faculty (SB), École Polytechnique Fédérale de Lausanne (EPFL), Valais/Wallis, Energypolis, CH-1951 Sion, Switzerland; ‡ Empa Materials Science & Technology, CH-8600 Dübendorf, Switzerland; § Institute of Materials, Ecole Polytechnique Fédérale de Lausanne (EPFL), CH-1015 Lausanne, Switzerland; ∥ Department of Chemistry, Graduate School of Science, Kyoto University, Kitashirakawa-Oiwakecho, Sakyo-ku, Kyoto 606-8502, Japan; ⊥ School of Environmental and Chemical Engineering, Shanghai University, 99 Shangda Road, Shanghai 200444, China

## Abstract

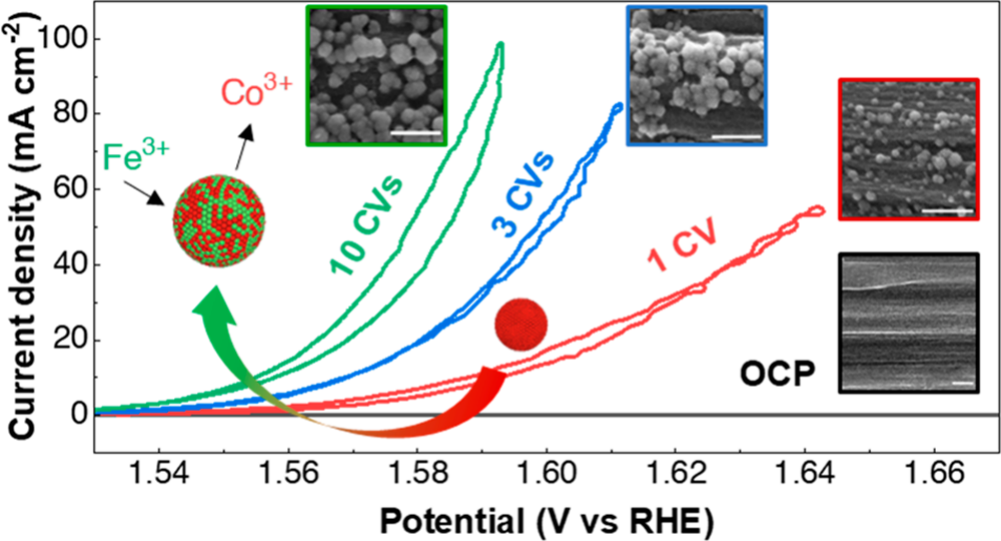

Ni- and Co-based catalysts with added Fe demonstrate promising activity in the oxygen evolution reaction (OER) during alkaline water electrolysis, with the presence of Fe in a certain quantity being crucial for their enhanced performance. The mode of incorporation, local placement, and structure of Fe ions in the host catalyst, as well as their direct/indirect contribution to enhancing the OER activity, remain under active investigation. Herein, the mechanism of Fe incorporation into a Co-based host was investigated using an in situ synthesized Co–Fe catalyst in an alkaline electrolyte containing Co^2+^ and Fe^3+^. Fe was found to be uniformly incorporated, which occurs solely after the anodic deposition of the Co host structure and results in exceptional OER activity with an overpotential of 319 mV at 10 mA cm^–2^ and a Tafel slope of 28.3 mV dec^–1^. Studies on the lattice structure, chemical oxidation states, and mass changes indicated that Fe is incorporated into the Co host structure by replacing the Co^3+^ sites with Fe^3+^ from the electrolyte. Operando Raman measurements revealed that the presence of doped Fe in the Co host structure reduces the transition potential of the in situ Co–Fe catalyst to the OER-active phase CoO_2_. The findings of our facile synthesis of highly active and stable Co–Fe particle catalysts provide a comprehensive understanding of the role of Fe in Co-based electrocatalysts, covering aspects that include the incorporation mode, local structure, placement, and mechanistic role in enhancing the OER activity.

## 1 Introduction

In alkaline water electrolysis, the oxygen evolution reaction (OER) is a sluggish multistep reaction involving four electrons that results in a considerable anode activation polarization. First-row transition metals such as Ni and Co have been demonstrated as promising candidates due to their low cost, abundance, and high OER catalytic activity in alkaline conditions.^1−4^ The presence of Fe in the structure has been reported to play a crucial role in enhancing the OER activity of Ni- and Co-based catalysts, regardless of their structure, for example, Ba_0.5_Sr_0.5_Co_0.2_Fe_0.2_O_3−δ_ (BSCF) of the perovskite family,^5^ Co_3–*x*_Fe_*x*_O_4_ with spinel structure,^6^ or Ni_0.75_Fe_0.25_OOH^7^ and Co_0.46_Fe_0.54_OOH^8^ of the layer-structure-type catalysts.

Extensive research has been conducted on the mechanism of Fe incorporation into Ni- and Co-based catalysts and its role in enhancing the OER activity. Conventionally, Fe is intentionally doped in the Ni and Co catalysts during the synthesis step by adjusting the composition of the precursor solution for wet chemical routes^9,10^ or that of the electrolyte bath for electrodeposition.^8,11^ Trotochaud et al. utilized grazing-incidence X-ray diffraction (GIXRD) to confirm that the cathodically deposited Ni_0.75_Fe_0.25_(OH)_2_ aged thin films in commercial KOH have a layered double hydroxide structure of Ni–Fe fougèrites.^12^ These minerals featured an extended *c*-axis compared to β-Ni(OH)_2_. The lattice change was attributed to the substitution of Ni^2+^ ions by Fe^3+^, resulting in an increased concentration of intercalated anions. Furthermore, Friebel et al., using operando X-ray absorption spectroscopy (XAS), found that Fe^3+^ cations do not intercalate between the γ-NiOOH sheets at Fe concentrations inferior to 25%.^7^ Instead, they tend to replace Ni^3+^ within a sheet due to the shorter Fe–O bond length in Ni_1–*x*_Fe_*x*_OOH compared to γ-FeOOH and similar to that of Ni–O. Computational calculations also indicated that Fe is the active site in Ni_1–*x*_Fe_*x*_OOH because it exhibits stronger adsorption of the OER intermediates compared to Ni. For the Co–Fe system, Burke et al. demonstrated that CoOOH provides an electrically conductive and stable host for the OER-active but poorly conductive FeOOH.^8^

Recently, an electrochemical approach using cyclic voltammetry (CV) in a Fe-containing electrolyte has been reported as an efficient way for adding Fe into Ni- and Co-based catalysts.^13,14^ Compared to the cathodic codeposition of the bulk Ni–Fe or Co–Fe catalyst, the incorporation of Fe directly via the OER alkaline electrolyte is usually termed incidental doping or Fe spiking. For this method, many studies have agreed that the addition of Fe in the catalyst structure is an irreversible process, as confirmed by Fe detection in the final catalyst. Deng et al. have shown, using operando atomic force microscopy (AFM), that exfoliated Ni(OH)_2_ transforms into NiOOH nanoparticles with a high surface area upon anodic oxidation, with FeOOH deposited as a separate phase in this porous structure.^15^ For Co-based catalysts spiked with Fe, Zhang et al. have demonstrated that intentional Fe incorporation has a stronger interaction with the CoOOH sheet compared to incidental Fe ions, which mainly localize at the edge of the hexagonal sheet and do not incorporate into the bulk structure, by analyzing their electrochemical profile.^16^ However, the lattice-scale structural modifications of the Ni- and Co-based catalysts induced by Fe have not been “visualized”, and the role of Fe in enhancing the OER activity remains elusive.

Here, we first synthesized a Co–Fe catalyst directly on top of the carbon rotating-disk electrode (RDE) by electrochemical cycling in a KOH electrolyte containing Co^2+^ and Fe^3+^ (in situ Co–Fe catalyst). This in situ synthesis enables the characterization of catalysts in their most natural state without the need for a polymer binder, which could potentially diminish surface hydrophilicity, hindering the electrolyte’s access to the catalyst or the release of oxygen. Moreover, it offers valuable insights into the preparation of nanostructures without the need of an additional exfoliation process^15^ or the use of organic agents,^17^ holding potential for applications in operando nanoscale characterization such as transmission electron microscopy (TEM) or AFM. In comparison to the highly inert bare glassy carbon, the in situ deposited Co–Fe catalyst exhibits an overpotential at 10 mA cm^–2^ of 319 mV and a Tafel slope of 28.3 mV dec^–1^. The electron microscopy and X-ray spectroscopy measurements reveal that the deposited Co–Fe catalyst has a Co-based host structure with a slightly larger lattice spacing compared to the deposited Co catalyst, induced by the Fe^3+^ substitution onto Co^3+^ sites. Moreover, operando optical spectroscopy and electrochemical quartz-crystal microbalance (EQCM) measurements demonstrate that the substituted Fe^3+^ cations reduce the transition potential from the CoOOH phase to the OER-active CoO_2_ phase.

## 2 Methodology

### 2.1 Preparation of the Electrolyte

The electrolyte was diluted from 50% potassium hydroxide (KOH, Carl Roth) with deionized water in order to obtain a 1 M KOH solution. To remove Fe contamination from the commercial KOH,^7,12,14^ the electrolyte was treated with nickel(II) hydroxide (Ni(OH)_2_, Fluka). A 1 g portion of Ni(OH)_2_ was added to 1 L of 1 M KOH, and the solution was stirred overnight. After the sedimentation of Ni(OH)_2_, the top solution was decanted and filtered through filter paper (Cytiva).

Neutral and acidic electrolytes were also prepared at a concentration of 1 M from potassium nitrate (KNO_3_, Alfa Aesar) and 65% nitric acid (HNO_3_, Sigma-Aldrich), respectively.

Different nitrate salts were prepared for the addition to the treated KOH electrolyte. Cobalt(II) nitrate hexahydrate (Co(NO_3_)_2_·6H_2_O, Sigma-Aldrich), iron(III) nitrate nonahydrate (Fe(NO_3_)_3_·9H_2_O, Sigma-Aldrich), copper(II) nitrate trihydrate (Cu(NO_3_)_2_·3H_2_O, Sigma-Aldrich), nickel(II) nitrate hexahydrate (Ni(NO_3_)_2_·6H_2_O, Sigma-Aldrich), and silver nitrate (AgNO_3_, Sigma-Aldrich) were prepared as 0.05 M solutions.

### 2.2 Preparation of the Electrode

For the activity test, the glassy carbon rotating disk electrode (GC-RDE) was polished with sandpaper of two different grit numbers, first with P500 and then with P1000 (VSM). After being rinsed thoroughly, the GC-RDE was polished again with a 0.05 μm polishing alumina suspension (BASi) on a polishing cloth (MicroCloth, Buehler). The RDE was rinsed again with Milli-Q-grade water and dried in air. The reference was a Ag/AgCl electrode, and the counter electrode was a Pt spring. Before use, the Pt counter electrode was soaked in HNO_3_ 25%, and then a blowtorch flame was applied to remove all contaminants or depositions from previous electrochemical reactions. Reference catalysts (IrO_2_||C and RuO_2_||C) were prepared by adding 10 mg of oxide catalysts (IrO_2_ (Sigma-Aldrich) or RuO_2_ (Sigma-Aldrich)), 15 mg of carbon black (Vulcan XC 72R, Fuel Cell Store), and 40 μL of Nafion (Nafion 117 containing solution, Sigma-Aldrich) into 1 mL of isopropanol. The mixture was then sonicated and drop-cast onto the GC-RDE with a surface loading of 0.3 mg cm^–2^ of oxide catalyst, 0.45 mg cm^–2^ of carbon black, and 0.06 mg cm^–2^ of Nafion.

For surface characterization, glassy carbon plates (Sigradur, HTW) and carbon papers (Toray, Alfa Aesar, and Sigracet 29 AA, FuelCellStore) were used. The electrochemical measurements were performed in an H-cell, which was composed of two compartments separated by a Nafion membrane. The working electrode was positioned close to the magnetic bar to reduce the effect of mass transport.

### 2.3 Electrochemical Characterization

A potentiostat (Metrohm Autolab PGSTAT204) was used for the electrochemical measurements. CV was performed with the conventional three-electrode chemical setup in 1 M KOH, with a pH of 14. We note that interaction between Co- and Ni-based catalysts and Fe contamination in commercial KOH was previously reported in many works.^7,12,14^ Thus, to avoid crossover effects created by the added Co(NO_3_)_2_·6H_2_O and Fe contamination in commercial KOH solution, the KOH used in this work, denoted as Fe-free KOH, was treated with Ni(OH)_2_. After treatment, the amount of Fe in commercial KOH was reduced from 55 to 5 ppb (Table S1).

Next, the potential was swept from 1.0 to 1.7 V, then back to 1.0 V vs the reversible hydrogen electrode (RHE) with a scan rate of 10 mV s^–1^. A rotating disk electrode system (RRDE-3A, ALS) was used to thoroughly degas the electrode surface during the CV cycle. The rotating speed of the RDE was fixed at 1600 rpm.

The electrochemically active surface area (ECSA) was estimated by performing CV cycles in a non-faradaic region over an interval of 100 mV at 9 scan rates: 10, 25, 50, 75, 100, 150, 200, 300, and 400 mV s^–1^. The charging current, *i*_c_, is related to the scan rate, θ, following the equation
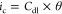
The ECSA is proportional to the double-layer capacitance, *C*_dl_, by *C*_s_^–1^, in which *C*_s_ is the specific capacitance of the sample:
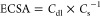
The typical value of *C*_s_ of a metal electrode in NaOH is reported to be 0.040 mF cm^–2^.^1,18^ The unit of the ECSA is cm^2^.

### 2.4 Materials Characterization

Scanning electron microscopy (SEM) images and corresponding energy-dispersive X-ray spectroscopy (EDX) elemental mapping were acquired on a ThermoFisher Teneo FE-SEM. High-resolution TEM (HR-TEM) images, high angle annular dark field (HAADF) images, and the corresponding EDX maps were obtained with a ThermoFisher Tecnai Osiris 200 kV TEM. Selected area electron diffraction (SAED) analyses were performed on a JEOL 2200FS 200 kV TEM. Electron energy-loss spectroscopy (EELS) was performed on a Titan Themis TEM (ThermoFisher Scientific, USA) equipped with a post column GIF Quantum ERS EELS spectrometer (Gatan, USA). The microscopy conditions for EELS acquisition were 300 kV, with a probe current of 0.07 nA, under scanning TEM (STEM) mode. The convergent and collection angles for EELS acquisition were 20 and 19.8 mrad, respectively. The energy resolution of the EELS data was determined by the full width at half-maximum of the zero-loss peak with the value of 1.1 eV using the dispersion condition of 0.1 eV per channel. Spectrum imaging was applied with the pixel time set at 0.1 s. Dual EELS was performed for all EELS acquisitions. Both low-loss and core-loss range were acquired to align the zero-loss peak position in the EEL data sets and deconvolve plural scattering in the core-loss spectra using Gatan Microscopy Suite (GMS).

XRD spectra were acquired with a Bruker D8 Advance system by using Cu Kα (λ = 1.54 Å) radiation. Inductively coupled plasma optical emission spectrometry (ICP-OES) was performed with an Agilent 5110 instrument.

X-ray photoelectron spectroscopy (XPS) was performed in an ultrahigh-vacuum spectrometer equipped with a VSW Class WA hemispherical electron analyzer. A Mg Kα X-ray source (1253.6 eV) was used as the incident radiation beam. The high-resolution spectroscopy was conducted with a constant pass energy of 22 eV, while survey scan was collected with a pass energy of 90 eV. The deconvolution of Co 2p and Fe 2p spectra were completed with CasaXPS software, while the attribution of binding energy to a specific metal phase was based on previous studies.^19−21^

Operando Raman spectroscopy was performed with our home-built Raman cell, which was also composed of three conventional electrodes. A glassy carbon plate was used as the working electrode for electrochemical measurements. An immersion objective (Leica, 63×) was used to send an incident beam and collect the scattered beam. Acquisition for low wavenumbers from 300 to 1200 cm^–1^ used a blue light with a wavelength of 457 nm, and that for high wavenumbers between 3000 and 4000 cm^–1^ used a red light of 633 nm. A constant potential between 1.1 and 1.7 V vs RHE was applied and held for 2 min before launching the Raman acquisition.

Operando EQCM measurements were performed with QCM922A (Seiko EG&G). Toray carbon paper was ground and mixed with isopropanol and Nafion and then drop-cast onto a Pt–quartz electrode. Ag/AgCl and Pt wire were used as the reference and counter electrodes, respectively. The standard resonance frequency of the Pt–quartz oscillator was 8.99 ± 0.03 MHz.

## 3 Results and Discussion

### 3.1 In Situ Synthesis of CoFe-Based Catalysts on Carbon Electrode and Their Characterization

**Figure 1:**
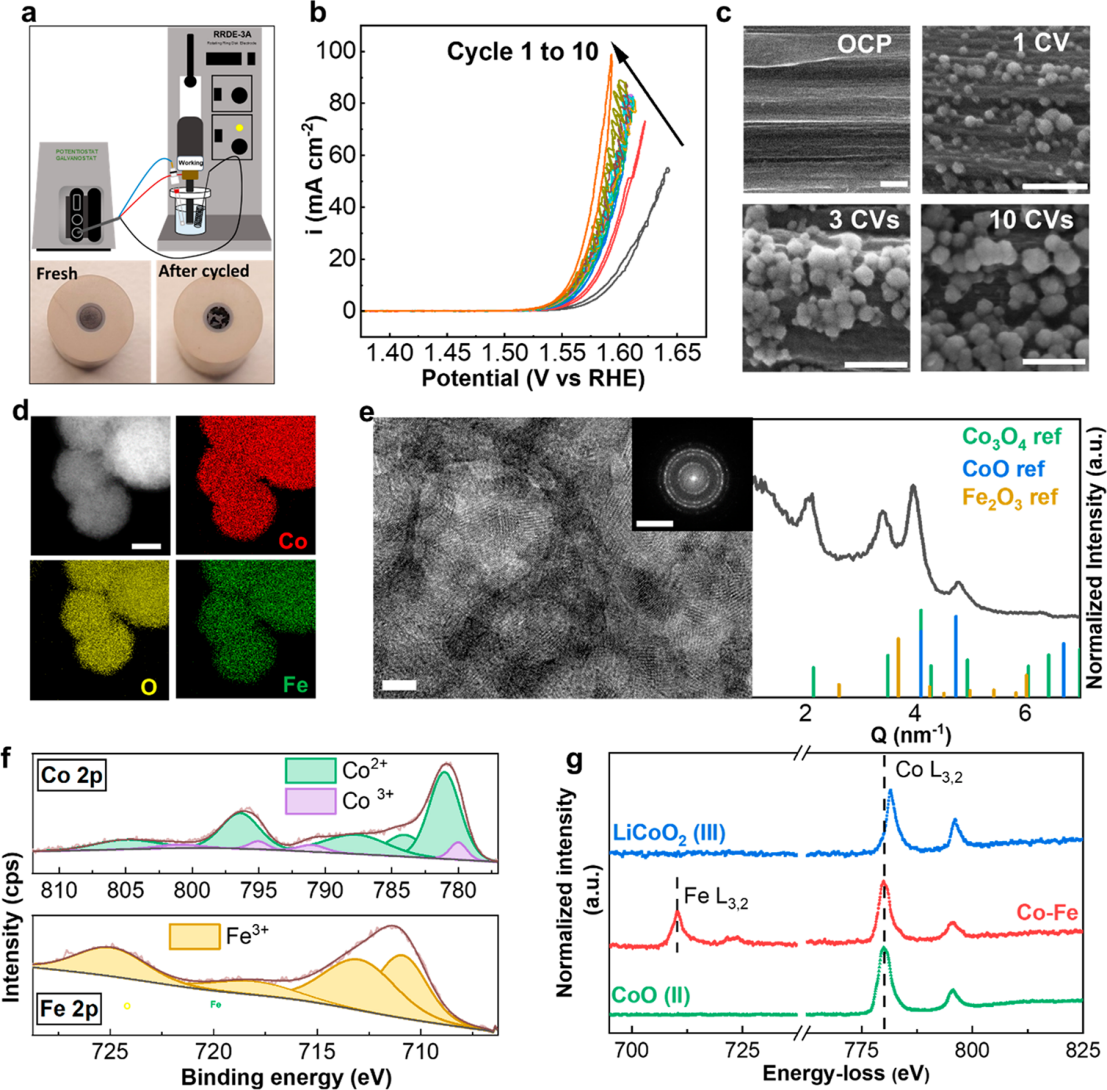
(a) Schematic of the RDE setup. (b) Evolution of the polarization curves of a GC electrode during 10 CV cycles in KOH-CoFe. The scan rate is 10 mV s^–1^, and the *iR* was corrected at 85%. (c) SEM images of the carbon paper at the OCP and after 1, 3, and 10 cycles of CV in KOH-CoFe. Scale bar: 500 nm. (d) HAADF-STEM image of in situ Co–Fe catalyst (after 10 CVs) and the corresponding elemental maps of Co and Fe. Scale bar: 50 nm. (e) BF-HRTEM image of the in situ Co–Fe catalyst (after 10 CVs). Scale bar: 5 nm. Inset: corresponding FFT; scale bar: 5 nm^–1^. Right: corresponding rotational average intensity. (f) XPS spectra for Co 2p and Fe 2p of in situ Co–Fe after 100 CVs (extended cycling is needed to collect a sufficient signal from the catalyst layer). (g) EEL spectra for Co L_3,2_ and Fe L_3,2_ of the in situ Co–Fe after 10 CVs. The spectra of CoO (II) and LiCoO_2_ (III) references are also shown.

The in situ synthesis of the CoFe-based catalysts, schematically demonstrated in Figure 1a, was performed in an RDE system. Co–Fe catalysts were precipitated on carbon electrodes by performing CV in an alkaline solution of 1 M KOH with 0.5 mM Co(NO_3_)_2_·6H_2_O and 0.2 mM Fe(NO_3_)_3_·9H_2_O (labeled as KOH-CoFe). The electrochemical cycling was done between 1.0 and 1.7 V vs RHE at a scan rate of 10 mV s^–1^ for 10 cycles, where an increase in the OER current density was observed from the first to the 10th cycle (Figure 1b). Compared to the fresh glassy carbon (GC) electrode, deposition of the Co–Fe catalyst was clearly observed after 10 CV cycles as shown in the photographs of the electrodes in Figure 1a. We note that similar catalyst deposition could be performed on various carbon paper supports (Figure S1). The surface of the carbon paper during the deposition process was investigated by SEM imaging. From a bare surface at open circuit potential (OCP, shown in Figure S2), spherical Co–Fe nanoparticles were formed after the first cycle (Figure 1c). The average size of these spherical catalysts was measured and found to be approximately 40 nm (Figures S3 and S4). From the 3rd to the 10th cycle, the newly formed Co–Fe particles increased both in size and population, as also demonstrated by the size distribution analysis in Figures S3 and S4. After 10 cycles, the carbon surface was almost fully covered by these Co–Fe particles (Figure 1c). Further experiments showed that Co–Fe catalysts with similar morphology were also deposited by applying a constant current density of 20 mA cm^–2^ (chronopotentiometry, CP) as shown in Figure S5, indicating that the in situ synthesis is an anodic deposition process. Unlike other transition metals such as Cu, Fe, Ag, and Ni, which do not show deposition upon application of anodic potential and consequently do not exhibit enhanced OER activity (Figure S6), the Co-based catalyst was the only one showing notable OER activity. When synthesizing by cycling the Co catalyst alongside other transition metals in KOH-CoCu, KOH-CoAg, KOH-CoNi, and KOH CoFe, we observed a decrease in overpotential at 10 mA cm^–2^ and a change in Tafel slope only when using KOH-CoFe (Figures 1 and S7). Additionally, no enhanced OER activity was observed when the GC electrode was cycled in KOH-NiFe, under experimental conditions similar to those of KOH-CoFe (Figure S8). This suggests that the CoFe-catalyst, deposited in situ, shows excellent OER activity and is particularly interesting for understanding the effects of Fe incorporation in this structure.

We further investigated the influence of pH on the in situ synthesis of the CoFe-based catalysts through CP activation in acidic and neutral environments. A constant current density of 20 mA cm^–2^ was first applied for 600 s on the GC electrode in KNO_3_–CoFe, followed by 10 CV cycles in 1 M Fe-free KOH. No activity enhancement was observed for the neutral solution (Figure S9a). Correspondingly, no particle deposition (or other change) was noticed on the carbon surface after the activation in neutral medium except for the residual salt (Figure S9b). An activation process in 1 M HNO_3_, similarly, did not modify the OER activity of the GC electrode (Figure S10), demonstrating that the deposition occurs only in an alkaline solution.

The chemical composition and elemental distribution of the Co–Fe catalysts obtained from the deposited layer on carbon paper were further investigated using STEM-EDX. Within a single Co–Fe catalyst particle, as shown in the HAADF-STEM image in Figure 1d, the EDX elemental maps reveal a homogeneous distribution of Co and Fe. STEM-EDX quantification indicates an overall 29 at. % Fe in the Co–Fe catalyst particle. We also note that the content of Fe in the Co–Fe catalyst particles can be adjusted by changing the concentration of Fe^3+^ in the electrolyte (Figure S11). The XRD analysis of the Co–Fe catalyst did not exhibit any characteristic peaks corresponding to Co- or Fe-based phases. Instead, only graphite-related features were observed, which can be attributed to the significant thickness of the carbon support material (Figure S12). As a result, the crystal structure of the Co–Fe catalyst was examined by analyzing the microdiffraction pattern obtained through electron diffraction in TEM. Figure 1e depicts a high-resolution bright-field TEM image of the Co–Fe catalyst after 10 CVs. The corresponding fast Fourier transform (FFT) of Co–Fe catalysts shows features of the polycrystalline phases of CoO and Co_3_O_4_ (Figure 1e), and no additional peaks associated with the Fe-rich phase are detected. The rotational average intensity of the selected area electron diffraction (SAED) pattern shows that the Co–Fe catalysts are predominantly composed of polycrystalline CoO, Co_3_O_4_, and amorphous CoFeO_*x*_ (Figure S13). We note that the peak corresponding to Co_3_O_4_ at 2.15 nm^–1^ was insignificant in the SAED patterns of the in situ Co–Fe catalyst but clearly observable in those of the Co catalyst (Figure S14), suggesting a weaker presence of Co^3+^ in the Co–Fe structure.

Moreover, XPS spectra indicate that Co in the Co–Fe catalyst is in a mixed oxidation state of Co^2+^ and Co^3+^, with Co^2+^ being the major component,^19,20^ while Fe is in the Fe^3+^ oxidation state^21^ (Figure 1f). Co L_3,2_ edge EEL spectra of the in situ Co–Fe catalysts are shown in Figure 1g. The Co L_3,2_ peak position matches the EELS of the CoO (II) reference, indicating that the valence of Co is predominantly +2 in the Co–Fe-based catalyst. The contribution of the +3 oxidation state with respect to the LiCoO_2_ (III) reference was almost unnoticeable for the Co–Fe catalyst in the EEL spectrum. To conclude, the in situ Co–Fe catalyst is composed of amorphous CoFeO_*x*_ and polycrystalline CoO and Co_3_O_4_ with mixed Co oxidation states (predominantly +2).

### 3.2 Identifying the Structural Phase of Fe in the Co–Fe Catalyst

**Figure 2:**
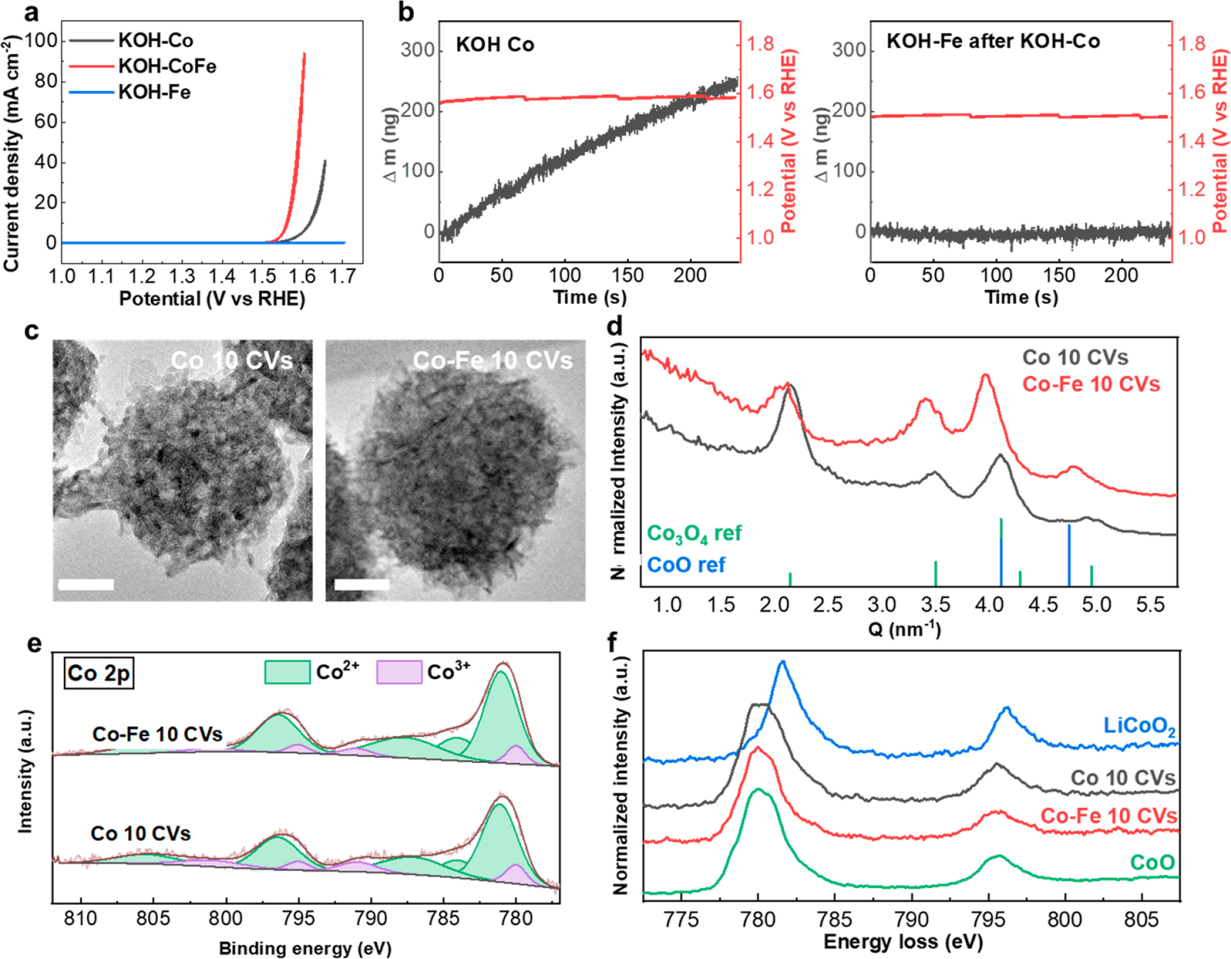
Comparison of the in situ Co and Co–Fe catalysts. (a) CV curves of the in situ CoFe, in situ Co catalysts, and glassy carbon cycled in KOH-Fe. The 10th cycle is plotted, with 85% of *iR* correction. The scan rate is 10 mV s^–1^. (b) Mass gain recorded during anodic polarization of carbon electrode in KOH-CO, at a current density of 25 mA cm^–2^. After that, no change in mass was observed during the anodic polarization of the electrodeposited Co in KOH-Fe, at a current density of 25 mA cm^–2^. (c) TEM images of in situ Co and in situ Co–Fe catalyst formed after 10 CVs in KOH-Co and KOH-CoFe, respectively. Scale bar: 20 nm. (d) Integrated intensity of the FFT pattern of in situ Co and in situ Co–Fe after 10 CVs (corresponding HR-TEM images in Figure S16). (e) XPS spectra for Co 2p of in situ Co–Fe and in situ Co catalysts formed after 100 CVs (extended cycling is needed to collect sufficient signal from the catalyst layer), with the corresponding peak deconvolution to Co^2+^ and Co^3+^. (f) EEL spectra for Co L_3,2_ of the in situ Co and Co–Fe catalysts after10 CVs. The spectra of CoO (II) and LiCoO_2_ (III) references are also shown.

To better understand the structural form of Co and Fe in the Co–Fe catalyst, we performed identical in situ synthesis separately in 1 M KOH + Co^2+^ (denoted as KOH-Co) or in 1 M KOH + Fe^3+^ (denoted as KOH-Fe) on GC electrodes. Interestingly, only the GC electrode cycled in KOH-Co showed an enhancement of the OER current density after 10 CV cycles (Figure 2a). The GC electrode cycled in KOH-Fe showed no activity enhancement compared to that cycled in Fe-free KOH. SEM images of the GC electrode showed that there was anodic deposition of Co particles after cycling in the KOH-Co electrolyte (Figure S15a), while no deposition occurred in the KOH-Fe electrolyte (Figure S15b). These results reveal that the anodic deposition takes place only with the presence of Co^2+^ in the electrolyte, and there is no deposited Fe-based phase in the Fe^3+^-containing electrolyte. It suggests that the in situ Co–Fe catalyst has a Co host structure, and Fe^3+^ incorporation occurs solely after the anodic deposition of the Co host phase.

Thus far, the route by which Fe is incorporated into the host structure remains unclear, whether it forms a separate second phase on the deposited scaffold or exists as a solid solution. Additionally, the specific location of Fe within the host structure is yet to be determined. With EQCM, we first evaluated the change in mass of the GC electrode by CV in KOH-Co and KOH-Fe electrolytes in sequence. When a current density of 25 mA cm^–2^ was applied to the carbon electrode in KOH-Co, we observed a gain in mass of the electrode corresponding to the anodic deposition of Co (Figure 2b). The potential recorded was 1.59 V versus RHE in KOH-Co. The electrode was then immersed in KOH-Fe and the same geometric current density was applied. In KOH-Fe, the recorded potential dropped from 1.59 V vs RHE in KOH-Co to 1.50 V vs RHE in KOH-Fe, confirming again the positive effect of Fe incorporation in enhancing the OER activity of the Co catalyst. Interestingly, there was no change in the mass of the deposited Co catalyst, indicating that no additional anodic deposition or insertion of an Fe-based phase onto the Co host structure occurred during cycling in KOH-Fe. The experimental design, along with the EQCM measurements, suggests that Fe is incorporated into the Co host structure by replacing Co at specific sites. Furthermore, the concentrations of Co^2+^ in the KOH-Fe solution were measured after performing CV or anodic CP experiments (Table S2). Following 10 CV cycles of the in situ Co in KOH-Fe, the Co^2+^ concentration in the KOH-Fe electrolyte rose from 15 to 65 ppb, demonstrating that Co is being released from the deposited catalyst into the solution. Similarly, after subjecting the in situ Co catalyst in KOH-Fe to 30 min of CP at 25 mA cm^–2^, we observed an increase in the Co^2+^ concentration in the KOH-Fe electrolyte from 20 to 50 ppb. The change in the Co^2+^ concentration in KOH-Fe following CV and anodic CP can be attributed to the leaching of Co from the deposited Co catalyst. These results provide additional evidence supporting the hypothesis of Fe-to-Co exchange.

Additionally, we compared the structure of Co and Co–Fe particles to further understand the specific location of Fe in the Co–Fe catalysts. The FFT shows that the reflections of Co catalysts are similar to those in the Co–Fe catalysts (Figure S14) with mixed Co oxide phases that include amorphous CoO_*x*_ and crystalline phases of rock salt CoO and spinel Co_3_O_4_. Bright-field TEM images show that both catalysts exhibit a spherical morphology (Figure 2c). HR-TEM images and the corresponding FFTs of the catalysts are shown in Figure S16. The crystal structure of Co–Fe catalysts resembles that of the Co catalysts, as suggested by the reflections in the FFT patterns occurring at similar spatial frequencies (Figure 2d). However, when overlapping the rotational average intensity of the two FFTs, we observed a slight shift of every peak to a smaller *Q* value, indicating a larger lattice parameter of approximately 3% in the case of Co–Fe (Table S3). The alteration in the lattice constant suggests the formation of a uniform solid solution consisting of Fe incorporated within the deposited Co catalysts. Additionally, at the particle level, we observed that the average size of Co–Fe spheres was larger than that of Co spheres following an equivalent number of cycles (Figures S3–S4, S17–S18). This observation aligns with the prior discovery made through operando AFM, which revealed a significant increase in particle height with the incorporation of Fe.^15^ Deconvolution of XPS spectra reveals that the ratios of Co^2+^ to Co^3+^ in in situ Co and Co–Fe catalysts are 4.83 and 8.62, respectively, meaning that there is less Co^3+^ in the Co–Fe catalyst (Figure 2e). This indicates that Fe^3+^ replaces Co^3+^ in the mixed oxide structure, therefore lowering the numbers of the Co^3+^ site. No shift in the main peaks of Co 2p was observed, suggesting that no change in coordination of Co sites takes place upon the addition of Fe. In the EEL spectra, despite the +2 oxidation state in both catalysts, the Co L_3,2_ fine structure of Co–Fe particles differs slightly from pure Co particles, as shown in Figure 2f. This is attributed to the modification of the electronic structure due to Fe incorporation in the cobalt host structure.

### 3.3 OER Activity and Stability of Co–Fe Catalysts

**Figure 3:**
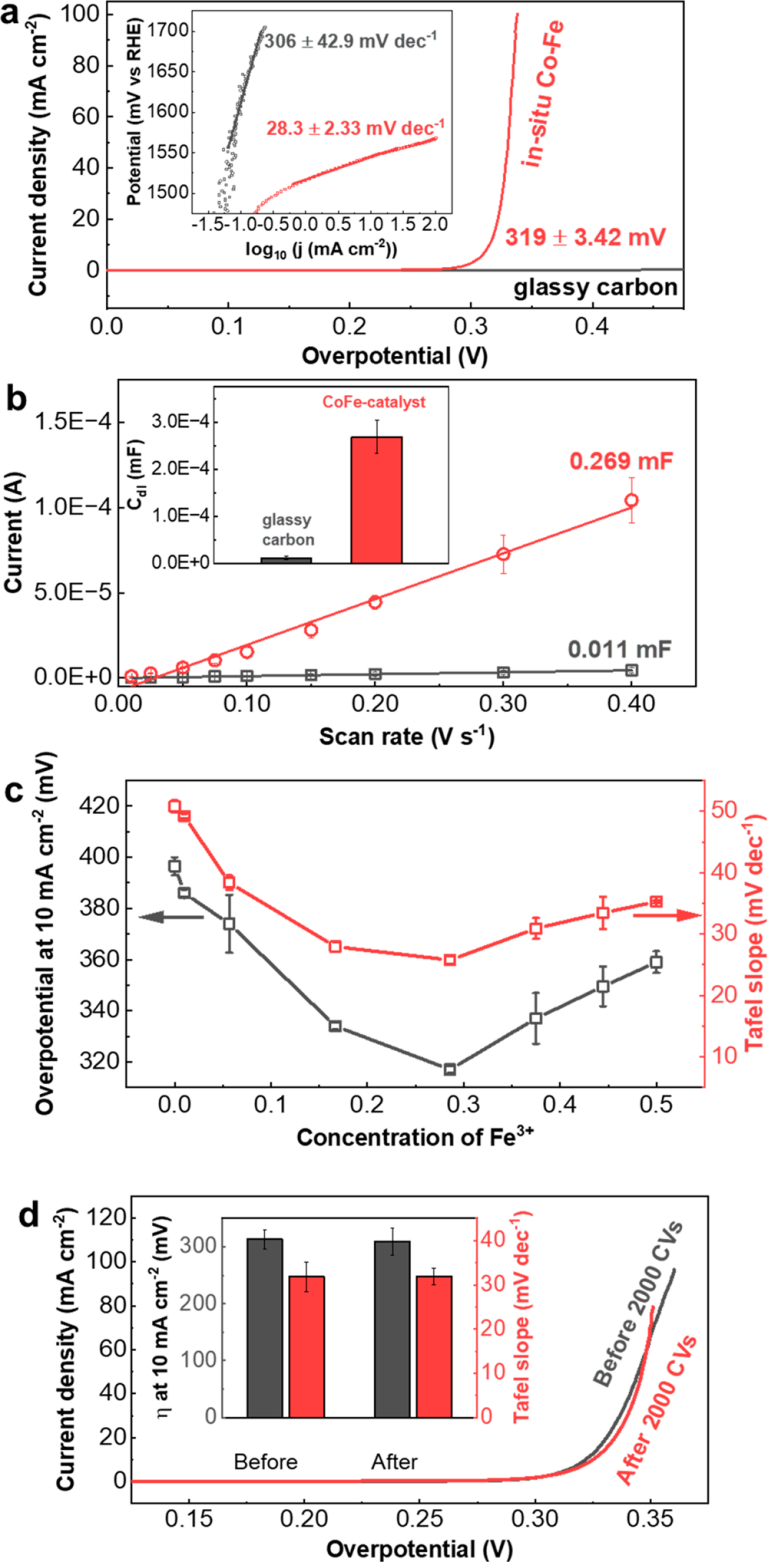
Activity for the OER of the in situ synthesized Co- and CoFe-based catalysts. (a) CV curves of GC in Fe-free KOH and in KOH-CoFe after 10 cycles. Inset: Tafel slopes. The scan range of the CVs was 1.0–1.7 V vs RHE, and the scan rate was 10 mV s^–1^. The CV curves and Tafel slopes were averaged over 10 individual measurements (Table S4). All the CV curves were corrected with 85% of *iR* drop. (b) ECSA of GC and in situ Co–Fe catalyst, acquired after 10 CVs in the electrolyte for synthesis. (c) Overpotential at 10 mA cm^–2^ and Tafel slope as a function of the Fe^3+^ concentration in the electrolyte (mM), obtained from 2 sets of measurements. (d) Activity for the OER before and after 2000 accelerated CVs in KOH-CoFe at 400 mV s^–1^ scan rate. The curve is plotted with CV measurements (10 CVs at 10 mV s^–1^) before and after the accelerated stability test.

Next, the OER activity of the in situ Co–Fe catalyst was evaluated (Figure 3a). The in situ synthesis was considered to be complete after 10 CV cycles, with full coverage of the carbon surface by the deposited Co–Fe catalyst (Figure 1c). The bare GC cycled in Fe-free KOH showed that it was inactive for the OER with only 0.23 mA cm^–2^ at 1.7 V vs RHE. Compared to the bare carbon surface, the in situ Co–Fe catalyst showed an overpotential at 10 mA cm^–2^ of 319 mV. Correspondingly, the Tafel slope dropped from 306 mV dec^–1^ for bare carbon to 28.3 mV dec^–1^ for the Co–Fe catalyst. The values were averaged from 10 sets of measurements, as summarized in Table S4. A variation range of ±10% for both the overpotential at 10 mA cm^–2^, and the Tafel slope emphasizes the high repeatability of our in situ synthesis method. The evolution of the OER activity of the in situ Co–Fe catalyst over 50 CV cycles at a scan rate of 10 mV s^–1^ is shown in Figures S19 and S20. The two kinetic parameters reached their stable value range after only 10 cycles, and they remained relatively stable from 10 cycles onward: between 315 and 328 mV for the overpotential at 10 mA cm^–2^ and between 28 and 32 mV dec^–1^ for the Tafel slope. Therefore, we emphasize the rapidity and practicality of the proposed in situ synthesis method to produce an OER-active catalyst.

The ECSA measurements of the in situ Co–Fe catalyst were performed after 10 CV cycles. The cycles at different scan rates in the non-faradaic region showed that the double-layer capacitance rose from 0.011 to 0.269 mF, corresponding to an increase of ECSA from 0.28 to 6.7 cm^2^ after only 10 CVs in KOH-CoFe, as shown in Figure 3b. To further evaluate the influence of the Fe/Co ratio on the OER activity, we gradually increased the concentration of Fe^3+^ and fixed the concentration of Co^2+^ at 0.5 mM (Figure S21). The content of Fe quantified by EDX represented as a function of the concentration of the Fe^3+^ precursor followed a linear relationship with a slope of 1.03 (Figure S11). This demonstrated that with our in situ synthesis method the content of Fe can be easily tuned by adjusting the ratio of Co^2+^ to Fe^3+^ in the KOH-CoFe solution. Figure 3c shows that both the overpotential at 10 mA cm^–2^ and the Tafel slope changed depending on the amount of Fe in the in situ synthesized Co–Fe catalyst. The two parameters followed an inversed volcano shape where the lowest values were obtained at an Fe^3+^ concentration of 0.2 mM, corresponding to approximately 28.6 at. % in the CoFe mixture (a trend obtained from 2 sets of measurements, Figure S22). This reverse volcano shape is very similar to previous findings for Ni–Fe bimetallic catalysts, in which the Ni–Fe film was prepared by cathodic electrodeposition in a mixed salt bath.^7,22^

The stability of the in situ Co–Fe catalyst was also evaluated in the same electrolyte where in situ synthesis occurred. The in situ Co–Fe catalyst was first formed with 10 CV cycles in KOH-CoFe with a scan rate of 10 mV s^–1^ and then underwent an accelerated stability test at a scan rate of 400 mV s^–1^. After 2000 accelerated CV cycles, the change of overpotential at 10 mA cm^–2^ was only 3 mV, from 313 to 310 mV, and the Tafel slope showed an increase of 0.1 mV dec^–1^ (Figure 3d). Even though the CV curve remained stable after 2000 accelerated cycles, we observed a change in size and distribution of the catalyst. Both the average size and the size distribution increased with the number of cycles, indicating a continuous nucleation of new particles, in parallel with the growth of the previously formed particles (Figures S3 and S4). The ECSA, on the other hand, primarily showed changes between the 1st and 10th cycles, with negligible evolution observed within the stability test range from the 10th to the 2000th cycle (Figure S23). The marginal 3 mV decrease of the overpotential agrees with the slight increase in ECSA between the 10th and 2000th cycle, which enhances the overall OER activity. In summary, the stability in OER activity can be assigned to the full geometrical coverage and the dynamic nucleation and growth of the in situ Co–Fe catalyst.

Similar measurements were performed on the in situ Co catalyst to evaluate its electrochemical performance for OER. We noticed an anodic shift of the Co^2+^/Co^3+^ redox peak of Co catalyst induced by doped Fe (Figure S24), which was previously assigned to the strong electronic interaction between Co and Fe.^8^ The overpotential at 10 mA cm^–2^, Tafel slope, ECSA, and stability of the in situ Co catalyst are presented in Table S5 and Figures S25–S29. The Co catalyst exhibited an overpotential at 10 mA cm^–2^ of 395 mV and a Tafel slope of 51.4 mV dec^–1^, with stability up to 2000 CV cycles. Its ECSA was 16-fold higher than bare glassy carbon yet only half that of the Co–Fe catalyst. The OER activity of the Co catalyst synthesized by our in situ method is among the best of Co-based compounds,^6,14,23−31^ as presented in Figure S30 and Table S6.

### 3.4 Operando Characterization of Co–Fe Catalyst at Anodic Polarization

**Figure 4:**
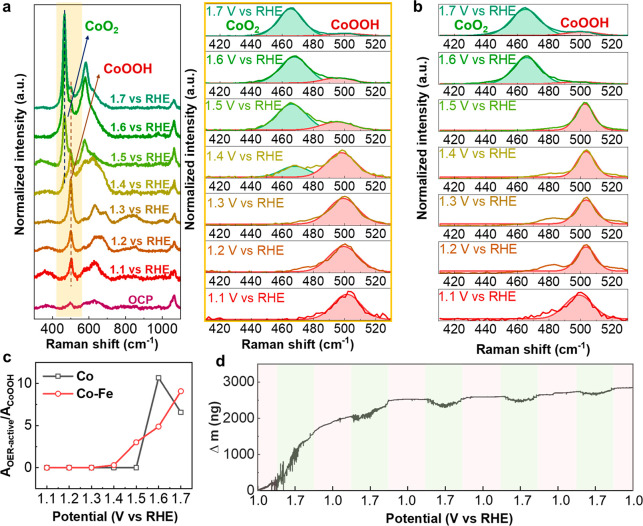
Tracking the catalyst evolution during OER. (a) Operando Raman measurements acquired on in situ Co–Fe catalysts at different applied potentials and the corresponding spectra deconvolution from 410 to 530 cm^–1^. Each spectrum was acquired 60 s after the application of the potential. (b) Operando Raman spectra of the in situ Co catalyst and its deconvolution. (c) Ratio of the area of OER-active CoO_2_ phase to that of CoOOH for in situ Co–Fe and Co, determined with Raman spectra deconvolution. (d) Change in mass of the in situ Co–Fe catalyst over 5 CVs. The drop of mass occurred when the potential went up from 1.4 to 1.7 and then down to 1.4 vs RHE. The red and green colors correspond to the phases plotted in (a).

Operando Raman measurements were performed to track the evolution of the surface structure of in situ Co–Fe and Co catalysts, at different applied potentials from 1.1 to 1.7 V vs RHE. In the in situ Co catalyst (Figure S31), we observed peaks at 503 and 686 cm^–1^ which have been previously reported to be characteristic for the E_g_ and A_1g_ vibrational modes of CoOOH^11,24,32,33^ (reference for peak positions summarized in Table S7). The small peak at 487 cm^–1^ is assigned to the glassy carbon surface, as observed in the Raman spectrum of the bare glassy carbon (Figure S32). For the in situ Co–Fe catalyst (Figure 4a), a similar phase of Co(Fe)OOH was observed in the entire range of applied potential, with the main peak at 503 cm^–1^ red-shifted to 497 cm^–1^ (Figure 4a). We also noticed a broad shoulder ranging from 600 to 700 cm^–1^ in the Co–Fe catalyst, instead of a sharp and intense peak at 686.5 cm^–1^ as observed in the pure Co catalyst. The presence of this shoulder and the red-shift of the main peak might be induced from a change in electronic structure due to the replacement of Fe^3+^ at Co^3+^sites.^34^ Starting from 1.4 and 1.6 V versus RHE for Co–Fe and Co catalysts, respectively, new peaks at 465 and 580 cm^–1^ were observed. Previous studies have assigned these peaks to the E_g_ and A_1g_ vibrational modes of the OER-active phase CoO_2_, prior to OER, which was formed after the redox reaction from Co^3+^ to Co^4+^, as summarized in Table S7.^24,33,35,36^ In order to understand the phase transition from CoOOH to the OER-active phase CoO_2_, we deconvoluted the Raman spectra (see Figure 4a,b for Co–Fe and Co catalysts) and plotted the ratio between their areas in Figure 4c. For the in situ Co catalyst, CoOOH is the only surface species from 1.1 to 1.5 V vs RHE, and the phase transition occurs only after 1.5 V vs RHE. In Co–Fe catalyst, the OER-active phase CoO_2_ appeared at 1.4 V vs RHE, and the area ratio between CoO_2_ and CoOOH increased from 1.4 to 1.7 V vs RHE, indicating that CoOOH is gradually replaced by the OER-active phase CoO_2_. This phase transition from CoOOH to OER-active CoO_2_ was also observed with EQCM. During the forward scan from 1.4 to 1.7 V vs RHE, we observed a decrease in mass of the Co–Fe catalyst which corresponds to a phase transition, as shown in Figure 4d. The change in mass was reversible when the potential was scanned backward from 1.7 to 1.4 V vs RHE. This transition was observed only between 1.55 and 1.7 V vs RHE for the in situ Co catalyst (Figure S33). At the potential range where the OER-active CoO_2_ phase was present, we also observed an increase in noise, which can be attributed to the generation of oxygen bubbles on the surface. The potential of transition from CoOOH to OER-active phase CoO_2_ identified by EQCM match those determined by operando Raman spectroscopy (red for CoOOH and green for CoO_2_ in Figure 4d). Therefore, operando Raman spectroscopy and EQCM demonstrate that the formation of the OER-active CoO_2_ phase takes place at 1.4 V vs RHE in Co–Fe catalyst and at 1.6 V vs RHE in the Co catalyst.

With Raman spectroscopy, we further investigated the hydroxyl stretching mode located between 3000 and 4000 cm^–1^ for the in situ Co–Fe and Co catalysts (Figures S34 and S35). The broad peak between 3100 and 3700 cm^–1^ was deconvoluted to three peaks. The first two peaks at low Raman shift were assigned to the two stretching vibrational modes of the OH band^37^ and the third peak at the highest shift to the M–OH bond.^38,39^ We observed a blue-shift in the M–OH peak of Co–Fe compared to that of the pure Co catalyst at all applied potentials, indicating that the M–OH bond is stronger in Co–Fe than Co (Figure S36). According to the volcano plot of the intrinsic activity as a function of M–OH bond strength proposed by Morales-Guio et al., the CoO_*x*_ is on the left branch while the FeO_*x*_ is on the right branch.^40^ For OER because OH^–^ adsorption is necessary in each single step, it must bind sufficiently strong to the metal to reach low overpotential.^41^ However, when the OH^–^ adsorption is too strong, the oxygenated species cannot be desorbed from the surface, leading to high overpotential.^42^ Thus, an intermediate M–OH bond strength is optimal for OER catalytic activity. In our in situ Co–Fe catalyst, the substitution of Fe^3+^ at Co^3+^ site helps increase the OH^–^ adsorption strength of Co, bringing the M–OH bond strength closer to the optimal value corresponding to the top of the volcano plot.

### 3.5 Incorporation Mechanism and Role of Fe in In Situ Co–Fe Catalyst

Based on the observations discussed in Section 3.2, it can be inferred that Fe^3+^ ions replace Co^3+^ ions within the Co-rich lattice, indicating a homogeneous incorporation of Fe by means of Fe-to-Co exchange rather than the formation of a separate phase. Therefore, the in situ synthesized Co–Fe catalyst can be defined as a Co-based structure with a uniform presence of Fe in its lattice. The catalyst retains the primary characteristics of anodically deposited Co oxides with a slightly enlarged lattice plane due to the incorporation of Fe.

**Figure 5:**
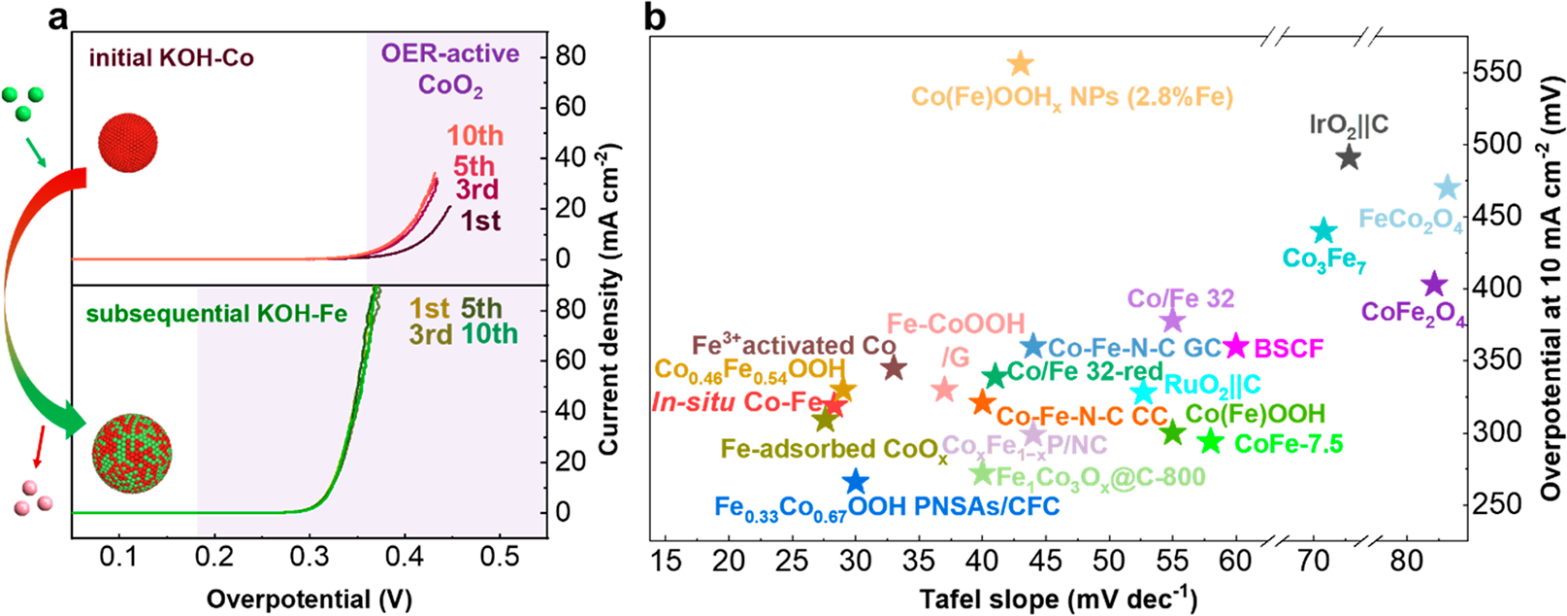
(a) Schematic of the proposed Fe^3+^ substitution on Co^3+^ sites for enhanced OER activity in the in situ Co–Fe nanocatalysts. (b) OER activity of the in situ Co–Fe catalyst compared with other reported CoFe-based catalysts. The details of the catalysts and electrolytes are summarized in Table S8.

By examining the electrochemical characteristics of the Co and Co–Fe catalyst, it is possible to predict the specific placement of Fe within the Co host structure. Previous studies of Fe-spiked NiOOH have shown that the incorporation of Fe at the edge of the NiOOH sheet was reflected in an increase in the activity without any change in the redox properties of the host phase. Upon cycling, the anodic shift in the redox peak suggests a gradual incorporation of Fe from the edge or defect sites into the bulk structure.^13^ During the cycling process in KOH-Co, we observed a gradual improvement in OER activity (Figure 5a), which can be attributed to the anodic deposition of the Co catalyst. At the same time, the area underneath the redox peak at −185 mV, corresponding to the Co^2+^/^3+^ redox wave,^16^ also increased, indicating a higher amount of deposited Co catalyst (Figure S37). The position of this peak remained unchanged throughout the process. Subsequently, when the deposited Co catalyst (10 CVs anodic deposition) was cycled in KOH-Fe, we noticed an increase in activity and an anodic shift of the redox peak by 25 mV, occurring spontaneously after the first cycle. This observation suggests an immediate incorporation of Fe into the bulk of the Co host structure. Throughout 10 CVs in KOH-Fe, both the OER activity (Figure 5a) and the redox peak (Figure S37) remained unchanged, indicating that the Fe remained stable within the bulk structure and there was no gradual evolution in its specific location during cycling. Based on the above discussion, we conclude that the in situ synthesis of the Co–Fe catalyst from an alkaline electrolyte containing Co^2+^ and Fe^3+^ ions involves a continuous process of Co deposition and Fe substitution onto the bulk Co host structure. During the first cycle, Co^2+^ is anodically deposited onto the carbon support to form a Co oxide host structure. This deposited structure is composed of amorphous CoO_*x*_, with crystalline nanoparticles of Co_3_O_4_ and CoO. After this first cycle, Fe^3+^ in the electrolyte is incorporated into the bulk structure of deposited Co oxide via substitution into a solid solution where Co^3+^ sites in the Co oxide host structure are replaced by Fe^3+^. With increased number of CV cycles, Co^2+^ continues to deposit, resulting in nucleation of new Co oxide nanoparticles on available carbon surface and growth of previously formed Co mixed-oxide nanoparticles. Simultaneously, Fe^3+^ keeps substituting Co^3+^ in the deposited Co-based host structure to form the final structure of the Co–Fe catalyst with uniform Fe distribution in the Co host structure and with larger lattice spacing (Figure 5a).

The resulting in situ Co–Fe catalyst is highly active for the OER due to an increase in both intrinsic and extrinsic activities. The enhanced extrinsic activity can be assigned to the increased ECSA of the in situ Co–Fe catalyst compared to that of the in situ Co catalyst. With the presence of Fe in the Co-based structure, the ECSA was boosted by a factor of 2 (Figure S28). The enhanced intrinsic activity with Fe incorporation is revealed by the lower Tafel slope of the Co–Fe catalyst in the ECSA-normalized current density (Figure S38). Indeed, operando Raman spectra and EQCM data support the fact that incorporation of Fe^3+^ in the mixed Co oxides reduces the formation potential for the OER-active CoO_2_ phase (Figure 5a), resulting to an enhancement of the intrinsic activity of the Co–Fe catalyst.

Figure 5b presents the overpotential at 10 mA cm^–2^ and the Tafel slope values of previously reported CoFe-based compounds compared with our in situ synthesized Co–Fe catalyst.^5,6,44−50,8,14,25,26,28,29,31,43^ The in situ catalyst synthesized herein outperforms a wide range of other CoFe-based catalysts and is among the best with other Co–Fe oxyhydroxides. Thus, with a simple method of adding Co^2+^ and Fe^3+^ into the electrolyte, we directly synthesized Fe-doped Co-based nanocatalysts on GC-RDE, with high intrinsic catalytic performance and high active surface area.

## 4 Conclusion

In conclusion, we have demonstrated the mechanism of Fe incorporation onto a Co host structure via the in situ synthesis of a highly active Fe-doped Co-based catalyst for the OER. It was shown that the in situ Co–Fe catalyst is comprised of an Fe solid solution phase within a Co host phase that is formed via anodic electrodeposition of Co^2+^ from the electrolyte. The Co base phase is composed of crystalline Co_3_O_4_, crystalline CoO, and amorphous CoO_*x*_ and exhibited an overpotential at 10 mA cm^–2^ of 395 mV and a Tafel slope of 54.1 mV dec^–1^. Our investigations reveal that the incorporation of Fe into the host structure occurred through the substitution of Fe^3+^ ions at Co^3+^ sites within the mixed Co oxides. This conclusion is supported by our analysis of the lattice structure and oxidation states of the Co–Fe catalyst as well as by in situ EQCM measurements. The Fe-doped Co catalyst exhibited further enhancement of the activity for OER, with the overpotential at 10 mA cm^–2^ and the Tafel slope reducing to 319 mV and 28.3 mV dec^–1^, respectively. This is due to the strengthened metal–OH bond in the Co base phase and increased electrochemically active surface area resulting from the presence of Fe. In addition to providing valuable insights into the mechanism and impact of Fe incorporation on enhancing the OER activity of Co-based catalysts, our research emphasizes the significance of in situ synthesis, which enables the characterization of the catalyst in its most natural state. This approach can be extended to other operando characterization methods of Co–Fe-based electrocatalysts, opening up possibilities for further exploration and understanding in this field.

## Supporting Information Available

Details regarding the elemental analysis of the electrolyte, post-mortem and operando structural characterizations of CoFe and Co catalysts, supplementary data on the OER activity, and electrochemical characterizations of the CoFe and Co catalysts (PDF)
