# Supplementary material for: Elucidating the Mechanism of Fe Incorporation in In Situ Synthesized Co–Fe Oxygen-Evolving Nanocatalysts
Source: J Am Chem Soc. 2023 Oct 20;145(43):23691–701. doi: 10.1021/jacs.3c08099 (PMC10623561; doi:10.1021/jacs.3c08099)
Supplement: Supplementary file 1 — ja3c08099_si_001.pdf [file ja3c08099_si_001.pdf]

Supporting Information for:

Elucidating the Mechanism of Fe Incorporation in In Situ Synthesized Co-Fe Oxygen-Evolving Nanocatalysts

Thi Ha My Pham,[a,b] Tzu-Hsien Shen,[c] Youngdon Ko,[a,b] Liping Zhong,[a,b] Loris Lombardo,[d] Wen Luo,[e] Satoshi Horike,[d] Vasiliki Tileli,[c,*] Andreas Züttel[a,b]

[a]Laboratory of Materials for Renewable Energy (LMER), Institute of Chemical Sciences and Engineering (ISIC), Basic Science Faculty (SB), Ecole Polytechnique Fédérale de Lausanne (EPFL) Valais/Wallis, Energypolis, Rue de l'Industrie 17, CH-1951 Sion, Switzerland

[b]EMPA Materials & Technology, CH-8600 Dübendorf, Switzerland

[c]Institute of Materials, Ecole Polytechnique Fédérale de Lausanne (EPFL), CH-1015 Lausanne, Switzerland

[d]Department of Chemistry, Graduate School of Science, Kyoto University, Kitashirakawa-Oiwakecho, Sakyo-ku, Kyoto 606-8502, Japan

[e]School of Environmental and Chemical Engineering, Shanghai University, 99 Shangda Road, Shanghai 200444, China

email: vasiliki.tileli@epfl.ch

Table S1. Fe concentration of the commercial and treated KOH, determined by ICP-OES at different wavelengths.

	Fe 234.350 nm (ppm)	Fe 238.204 nm (ppm)	Fe 239.563 nm (ppm)	Fe 259.940 nm (ppm)	Average
**Commercial KOH 1 M**	0.06	0.06	0.06	0.04	0.055
**Treated KOH 1 M**	0.00	0.00	0.02	0.00	0.005

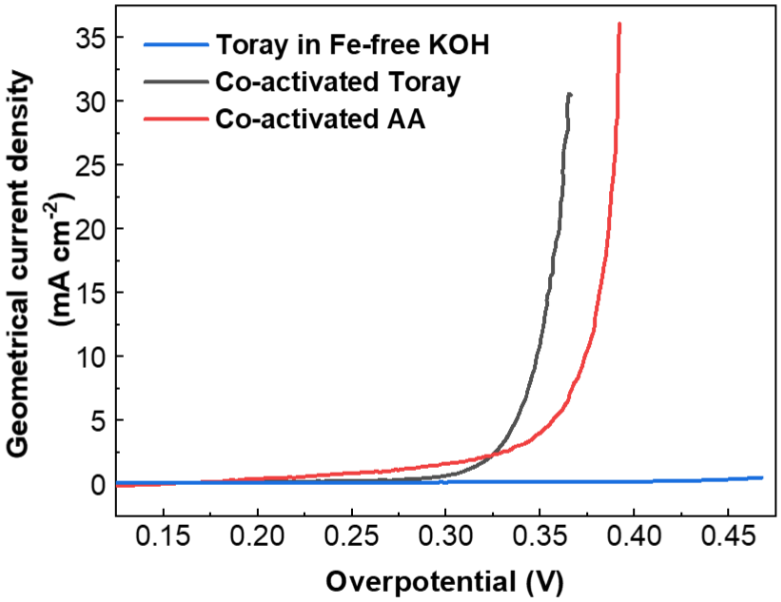

Figure S1. Geometrical current density as a function of overpotential of two carbon papers Toray and Sigracet 29 AA activated in KOH-Co.

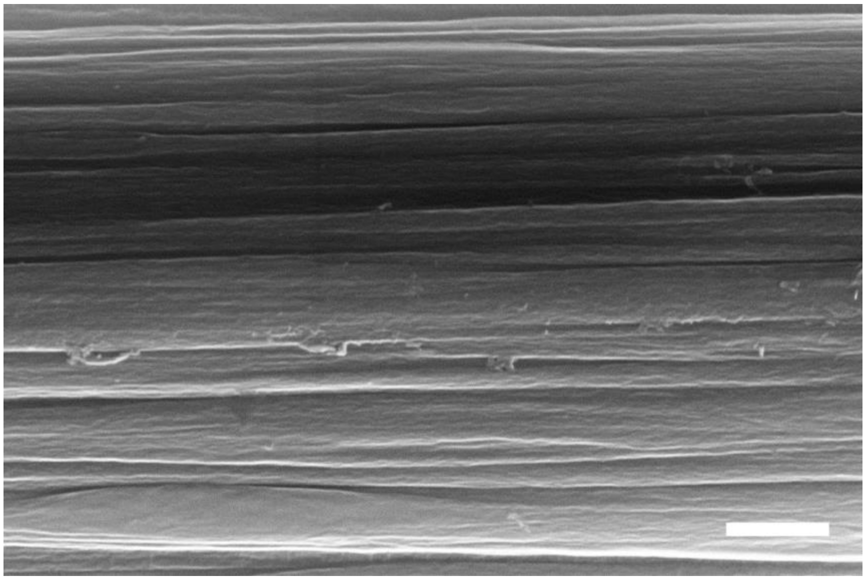

Figure S2. SEM image of the carbon paper after 10 CVs in Fe-free KOH. No deposited catalyst has been observed. Scale bar: 1 µm.

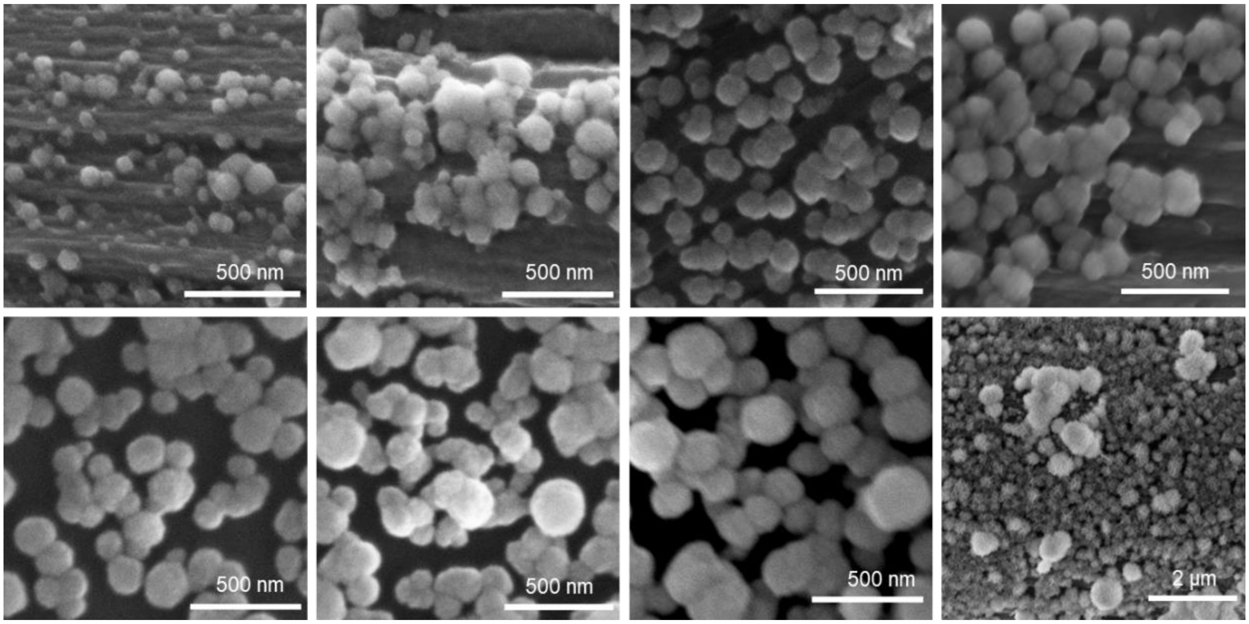

Figure S3. SEM images of the spherical particles formed on Toray carbon paper after different number of cycles in KOH-CoFe (from left to right): 1, 5, 10, 30, 50, 100, 300 and 3000 cycles.

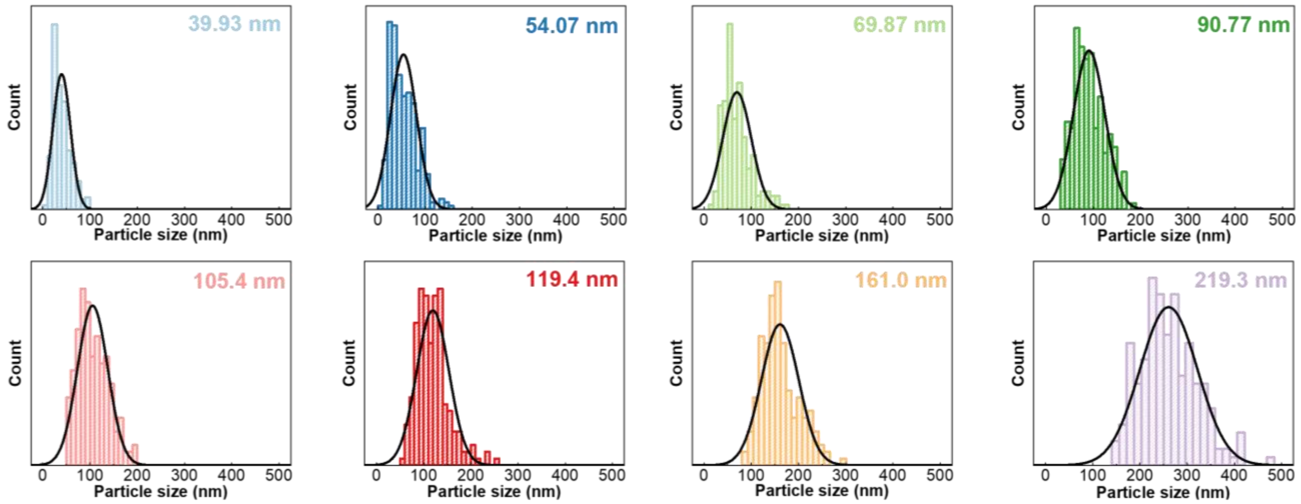

Figure S4. Size distribution of the spherical particles formed on Toray carbon paper after different number of cycles in KOH-CoFe (from left to right): 1, 5, 10, 30, 50, 100, 300 and 3000 cycles.

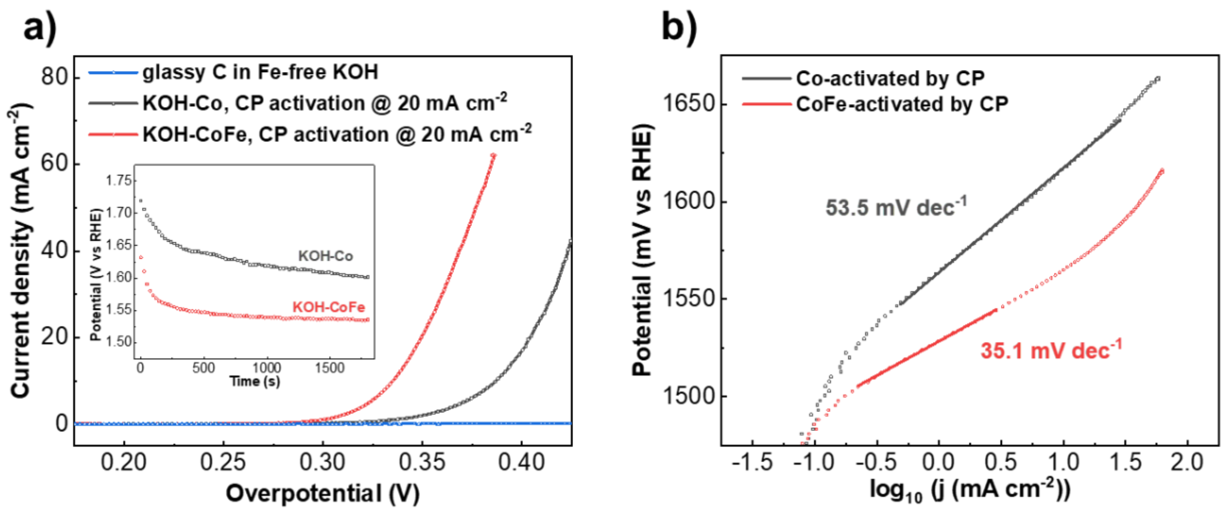

Figure S5. Activity for OER of different CP activation of GC-RDE in KOH-Co and in KOH-CoFe **a)** Current density as a function of overpotential. Inset: Evolution of the potential as a function of time at 20 mA cm^{-2}. **b)** corresponding Tafel slopes.

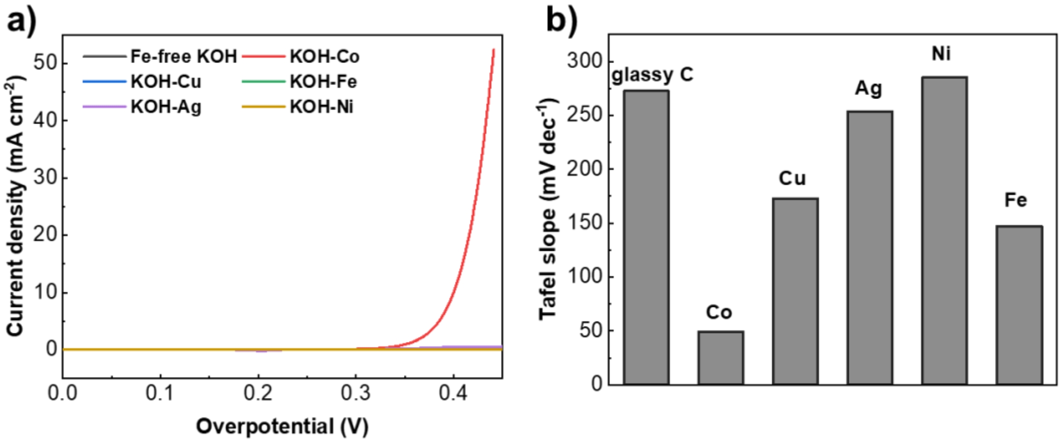

Figure S6. GC-RDE electrode cycled in different electrolytes: Fe-free KOH, KOH-Co, KOH-Cu, KOH-Fe, KOH-Ag and KOH-Ni, at a scan rate of 10 mV s^{-1}. a) Current density as a function of overpotential. b) Summary of the Tafel slopes.

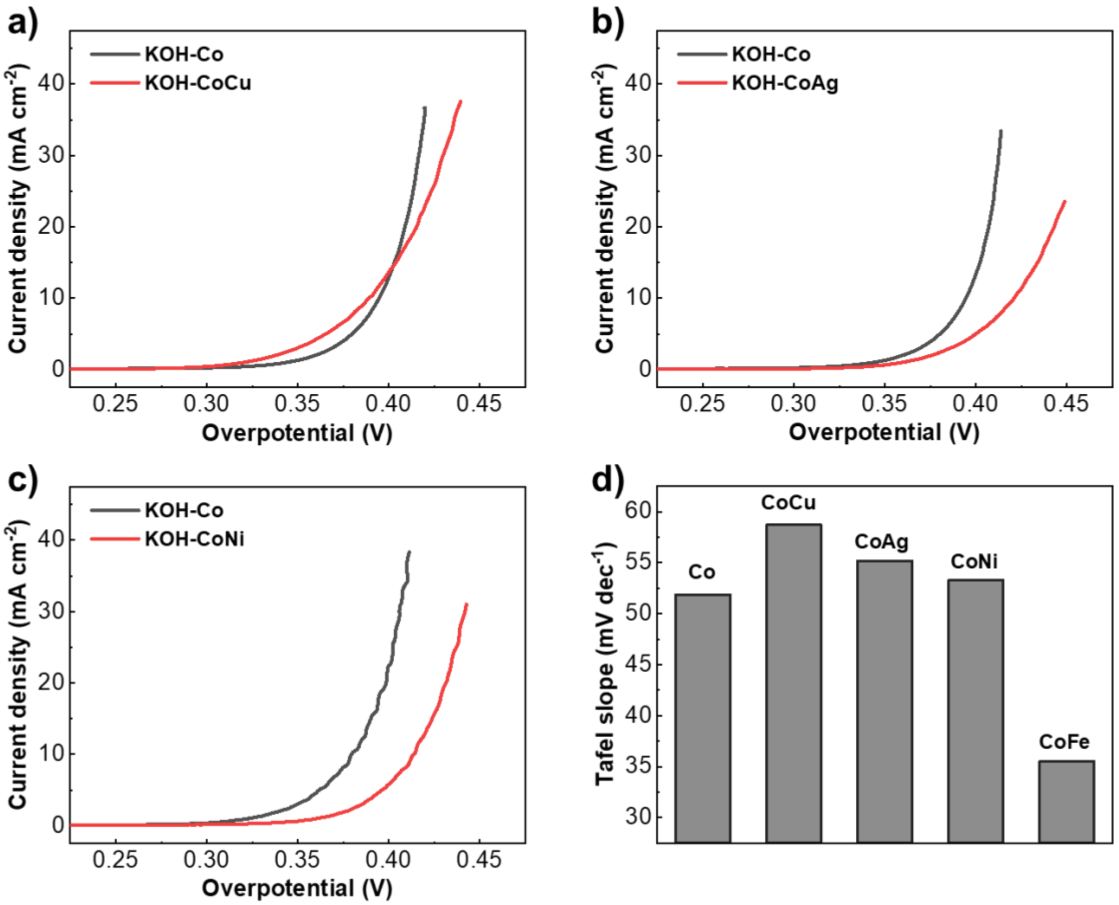

Figure S7. Deposited Co-catalyst cycled in different electrolytes, at a scan rate of 10 mV s^{-1}. a) KOH-CoCu, b) KOH-CoAg, c) KOH-CoNi. d) Summary of the Tafel slopes.

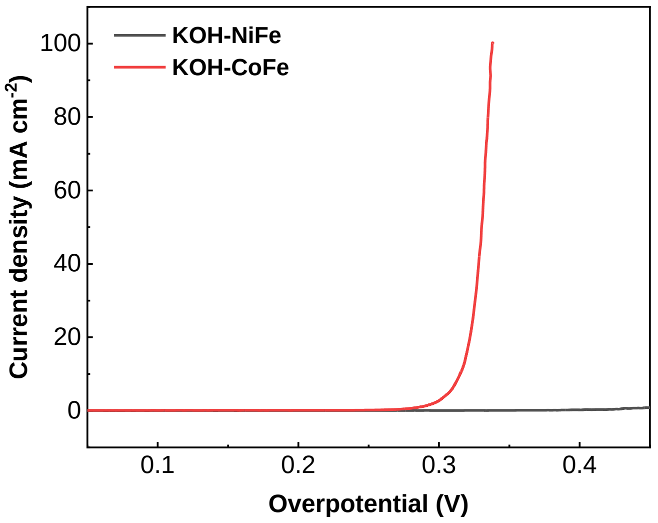

Figure S8. GC-RDE electrode cycled in KOH-NiFe, composed of Fe-free KOH + 0.5 mM Ni^{2+} + 0.2 mM Fe^{3+}, under similar electrochemical conditions as KOH-CoFe.

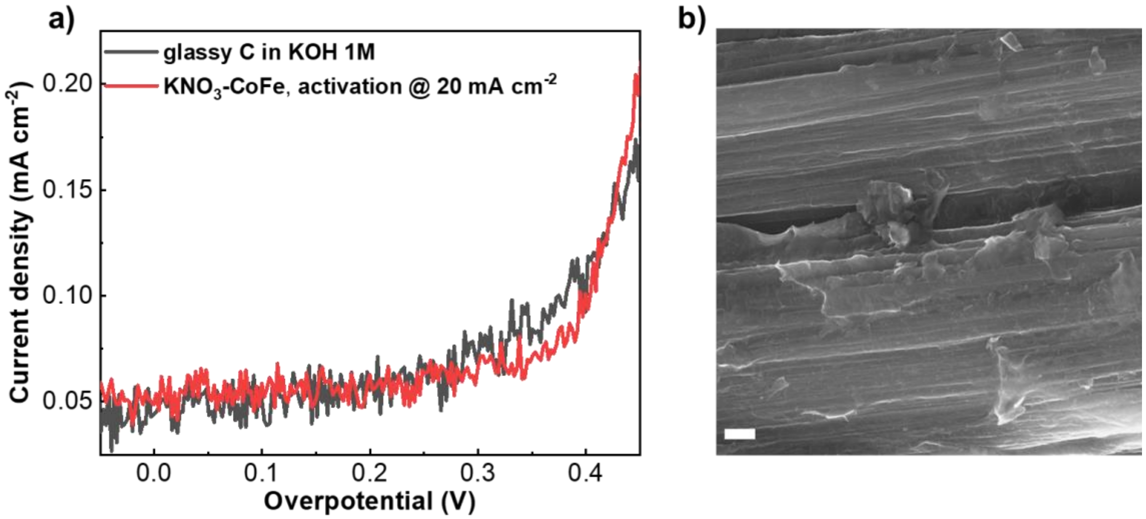

Figure S9. Anodic CP activation of the GC-RDE in KNO_3 1 M with the presence Co^{2+} and Fe $^{3+}$. a) Current density as a function of overpotential. b) SEM image of the Toray carbon paper after CP activation in neutral conditions. Scale bar: 1 µm.

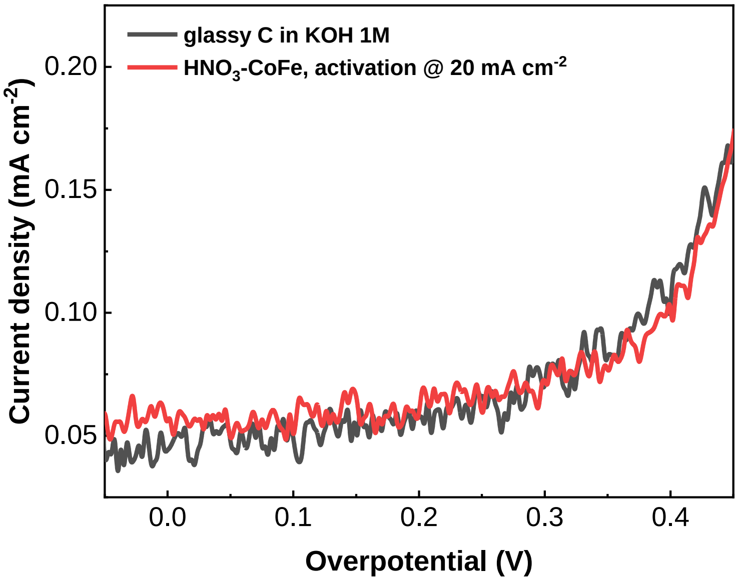

Figure S10. Anodic CP activation of the GC-RDE in HNO_3 1 M with the presence of Co^{2+} + Fe^{3+}.

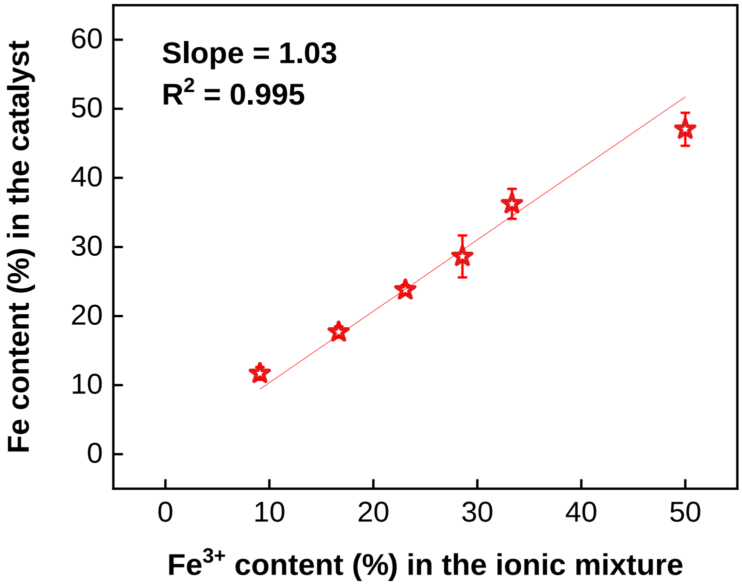

Figure S11. The correlation curve between the content of Fe in the catalyst determined by EDX and the concentration of Fe^{3+} in the ionic mixture.

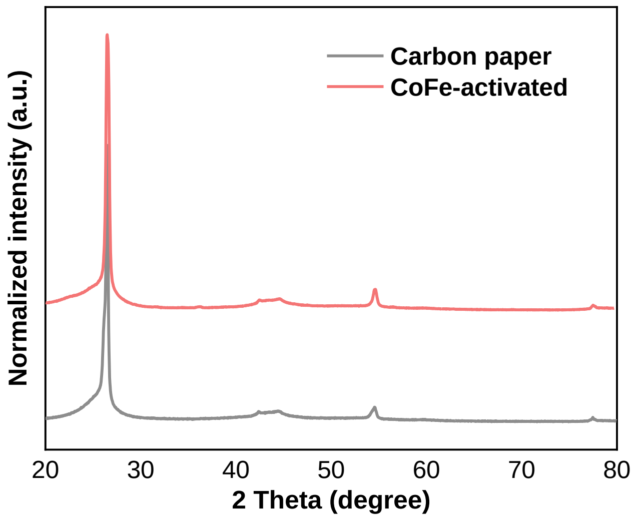

Figure S12. XRD pattern of the dry Toray carbon paper and that activated in KOH-CoFe.

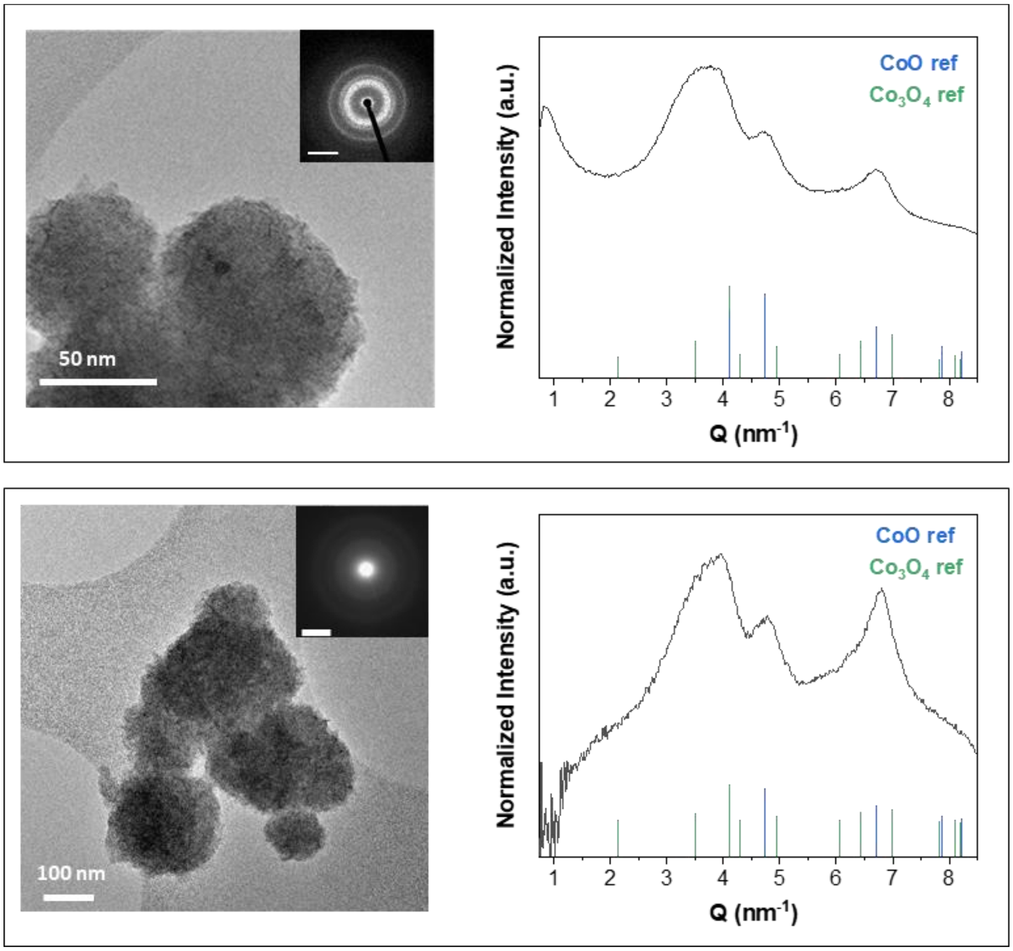

Figure S13. BF-TEM image, SAED patterns (scale bar: 5 nm^{-1}) and the corresponding integrated intensity of in situ Co-Fe catalyst, acquired at two different regions.

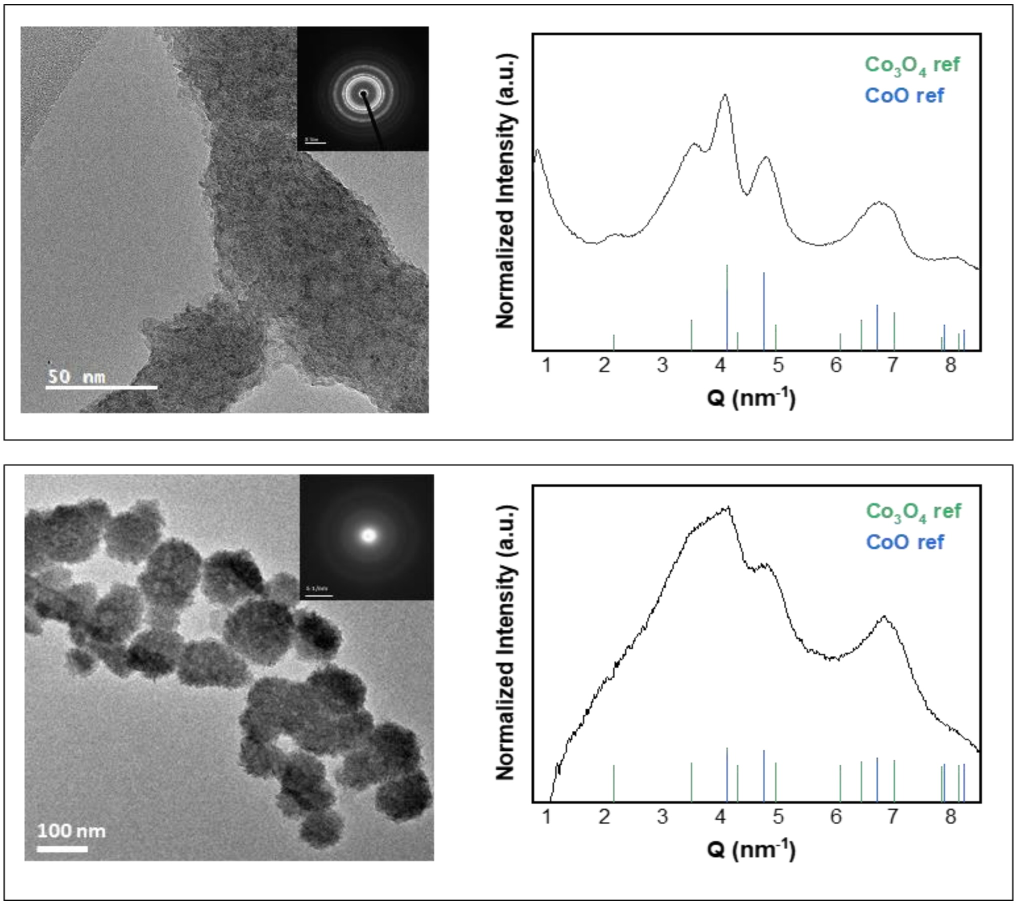

Figure S14. BF-TEM image, SAED patterns and the corresponding integrated intensity of in situ Co catalyst, acquired at two different regions.

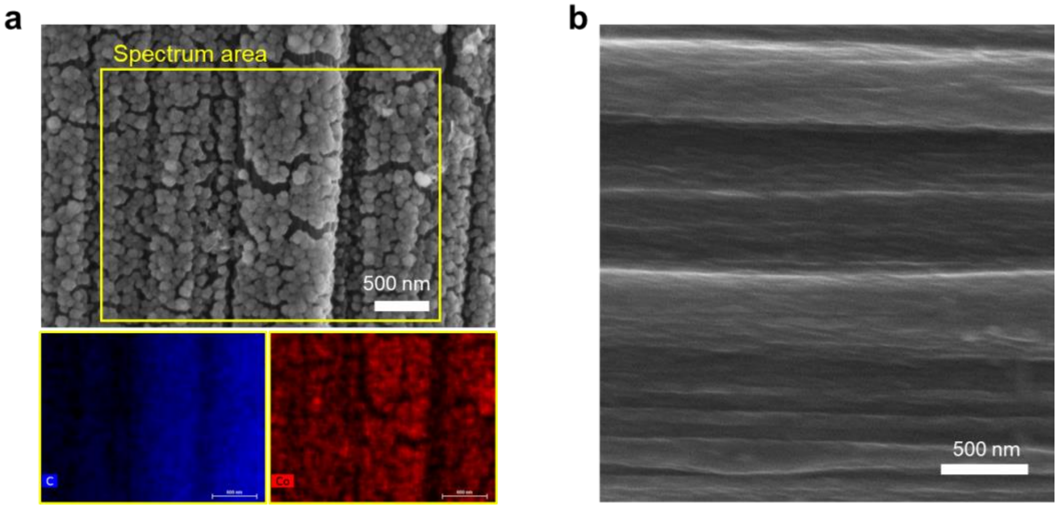

Figure S15. a) SEM image and the corresponding EDX of the in situ Co catalyst, formed after 10 CVs of Toray carbon paper in KOH-Co. b) SEM image of Toray carbon paper after 10 CVs in KOH-Fe. No deposition was observed.

Table S2. Concentration of Co in KOH-Co and subsequential KOH-Fe, determined with ICP-OES. The in situ Co catalyst was first deposited in KOH, by 10 CVs and by 30 minutes of CP at 25 mA cm^{-2}. After deposition, the Co-catalyst underwent similar CV and anodic CP tests in KOH-Fe. Upper line = initial concentration in Co, lower line = Co concentration after the electrochemical tests.

Co concentration (ppm)	10 CVs	CP 30 min at 25 mA cm^{-2}
**Deposition in KOH-Co**	10.79476	12.61379
	6.109813	10.10931
**Subsequential cycling in KOH-Fe**	0.017138	0.019824
	0.064708	0.050088

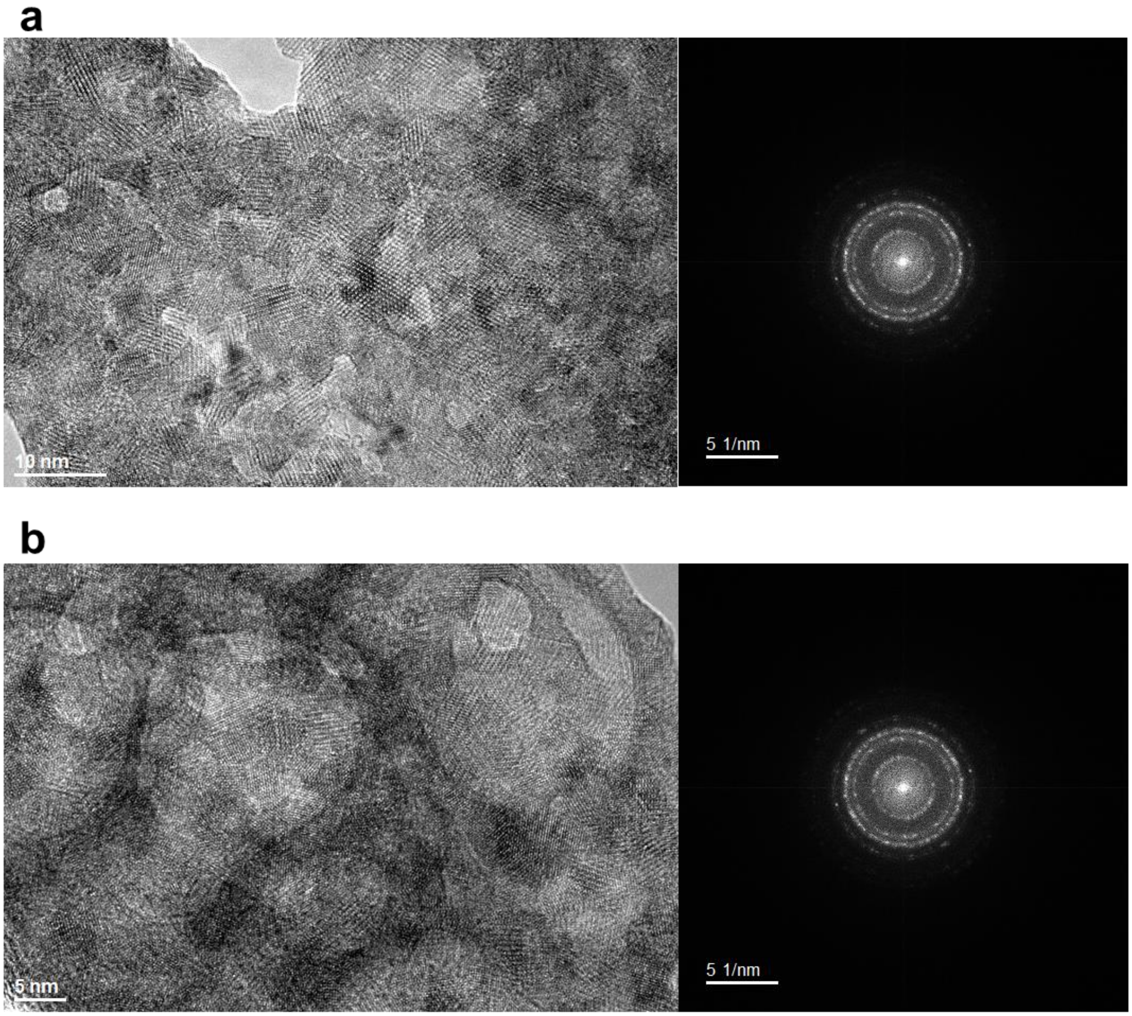

Figure S16. HR-TEM images and the corresponding FFT of a) in situ Co catalyst. b) in situ Co-Fe catalyst.

Table S3. Q-value and corresponding lattice parameters of in situ Co and Co-Fe catalyst, extracted from integrated intensity of the FFT in **Figure S16.** In the last column: calculated expansion of the lattice parameter in Co-Fe with respect to that of Co catalyst.

a_{Co} (nm^{-1})	$a_{Co\text{-}Fe}$(nm^{-1})	d_{Co}(Å)	$d_{Co\text{-}Fe}$(Å)	Expansion (%)
**2.148**	2.089	4.656	4.786	2.805
**3.489**	3.407	2.866	2.935	2.422
**4.096**	3.964	2.442	2.523	3.330
**4.941**	4.790	2.024	2.088	3.172

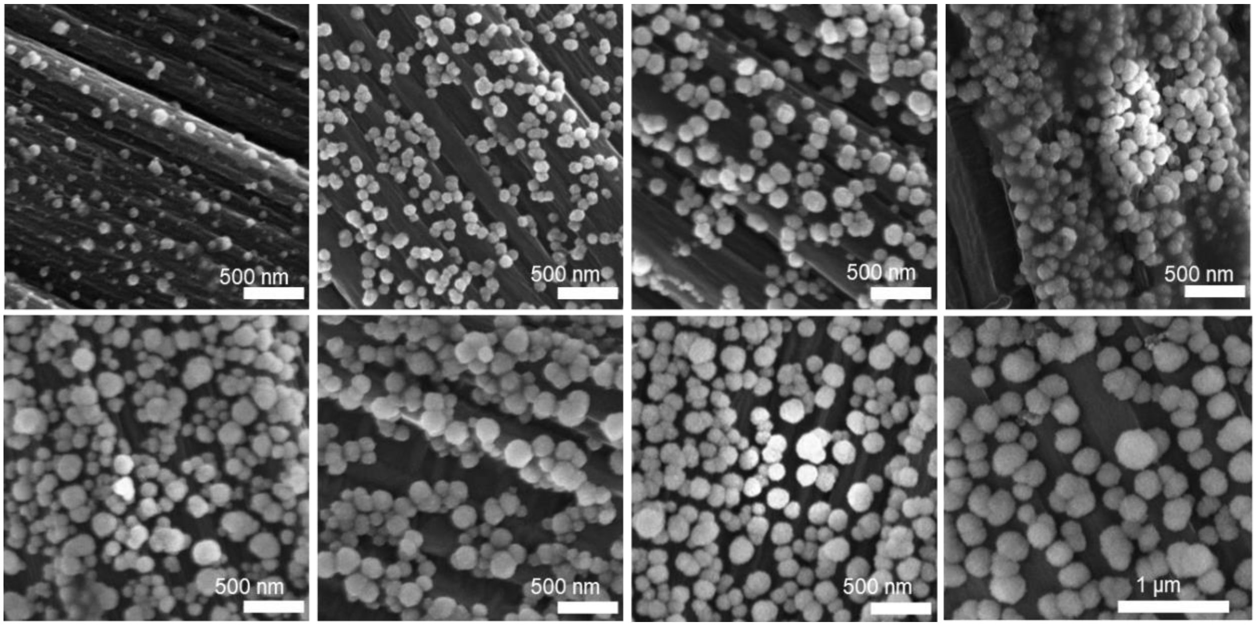

Figure S17. SEM images of the spherical particles formed on Toray carbon paper after different number of cycles in KOH-Co (from left to right): 1, 5, 10, 30, 50, 100, 300 and 3000 cycles.

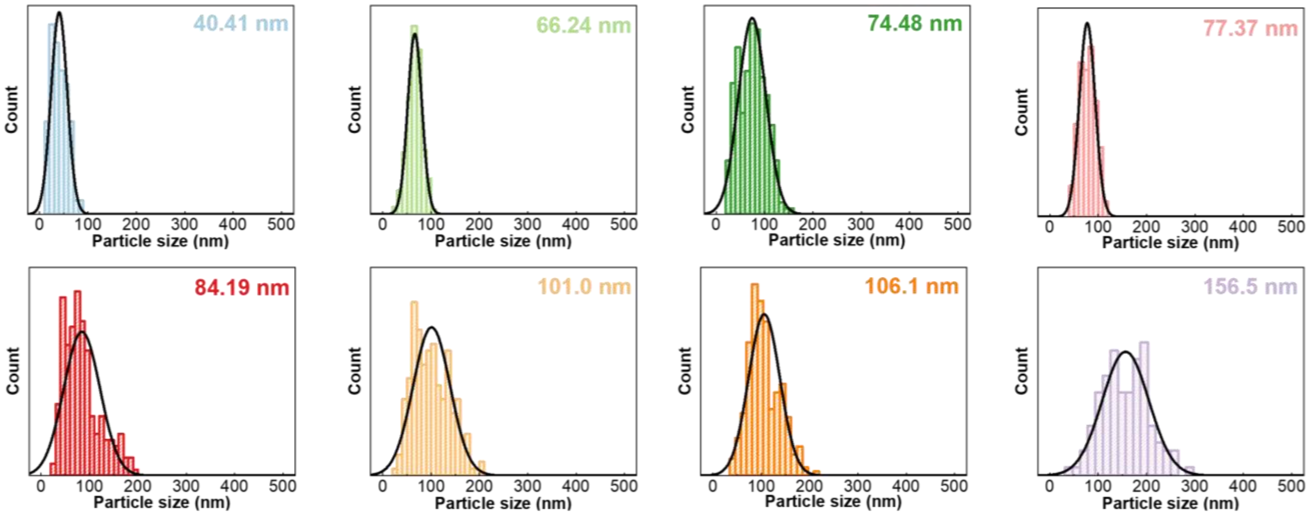

Figure S18. Size distribution of the spherical particles formed on Toray carbon paper after different number of cycles in KOH-Co (from left to right): 1, 5, 10, 30, 50, 100, 300 and 3000 cycles.

Table S4. Summary of the overpotential at 10 mA cm^{-2} and the Tafel slope of 10 different samples, measured after 10 CVs in KOH Co-Fe.

	**Overpotential at 10 mA cm^{-2} (mV)**	**Tafel slope ($mV\ dec^{-1}$)**
**1**	316	26.2
**2**	319	26.72
**3**	320	28.33
**4**	322	28.4
**5**	326	33.5
**6**	319	27.5
**7**	315.5	27.3
**8**	322.6	28.46
**9**	315.5	30.37
**10**	318	25.3
**Average**	319.4 ± 3.42	28.2 ± 2.33

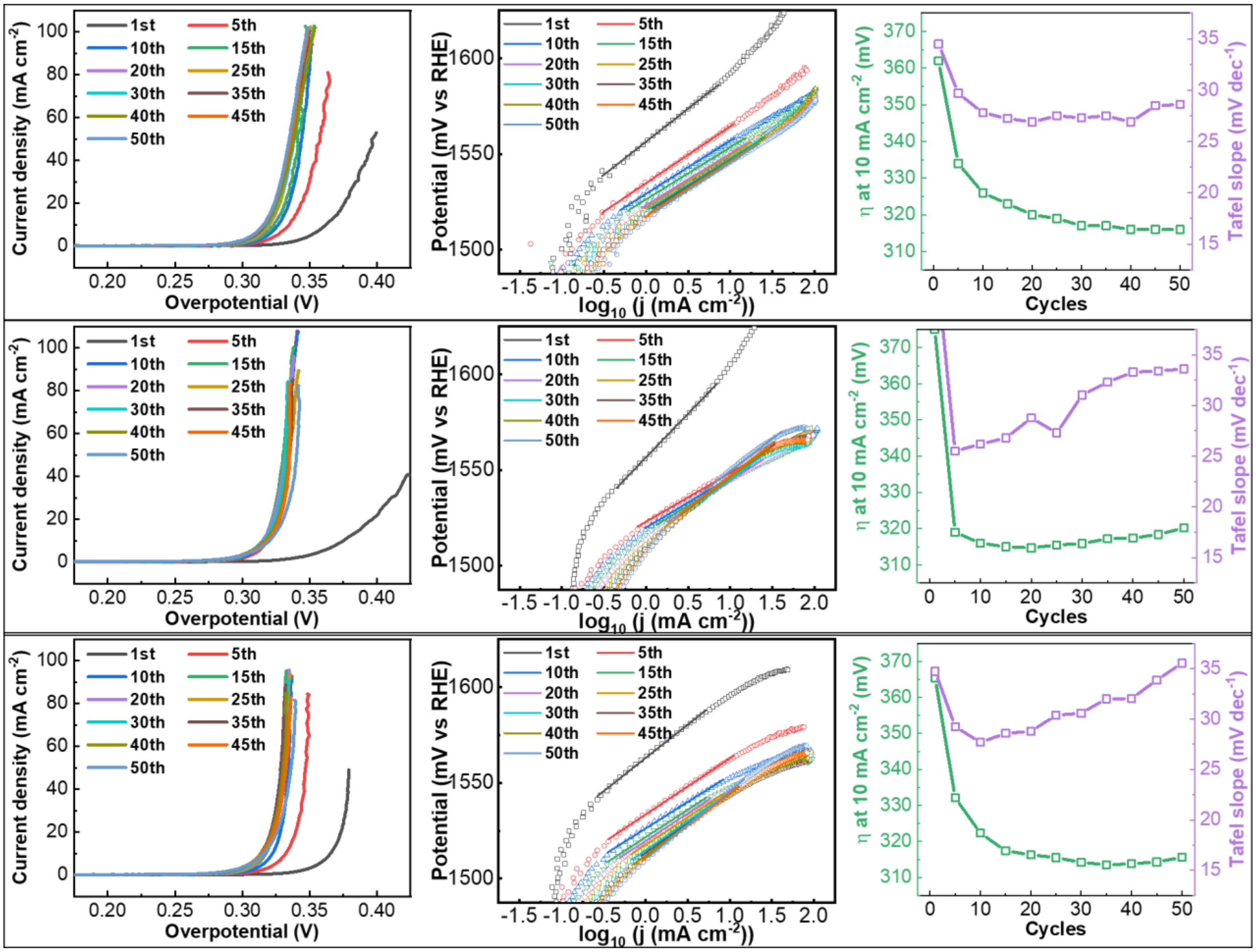

Figure S19. Evolution of the CV scan at 10 mV s^{-1} over 50 cycles in KOH-CoFe. Each sub-figure represents a set of measurements. From left to right: current density as a function of overpotential, Tafel slope, overpotential at 10 mA cm^{-2} and Tafel slope over as a function of number of cycles.

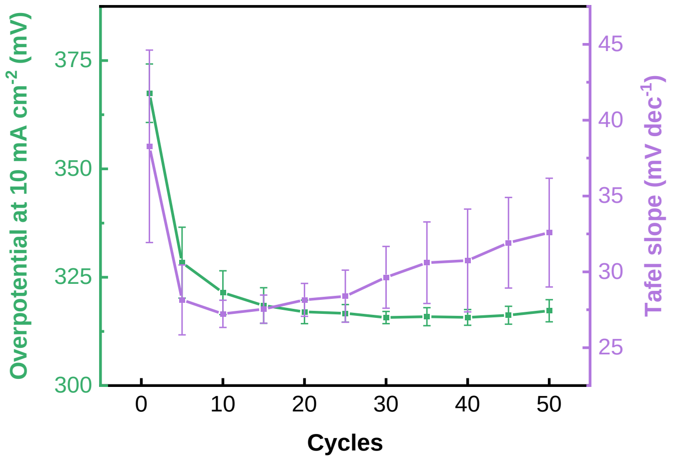

Figure S20. Evolution of the overpotential at 10 mA cm^{-2} and the Tafel slope over 50 CVs in KOH-CoFe. The scan rate was 10 mV s^{-1}. The error bar was averaged from 3 sets of measurements in **Figure S19**.

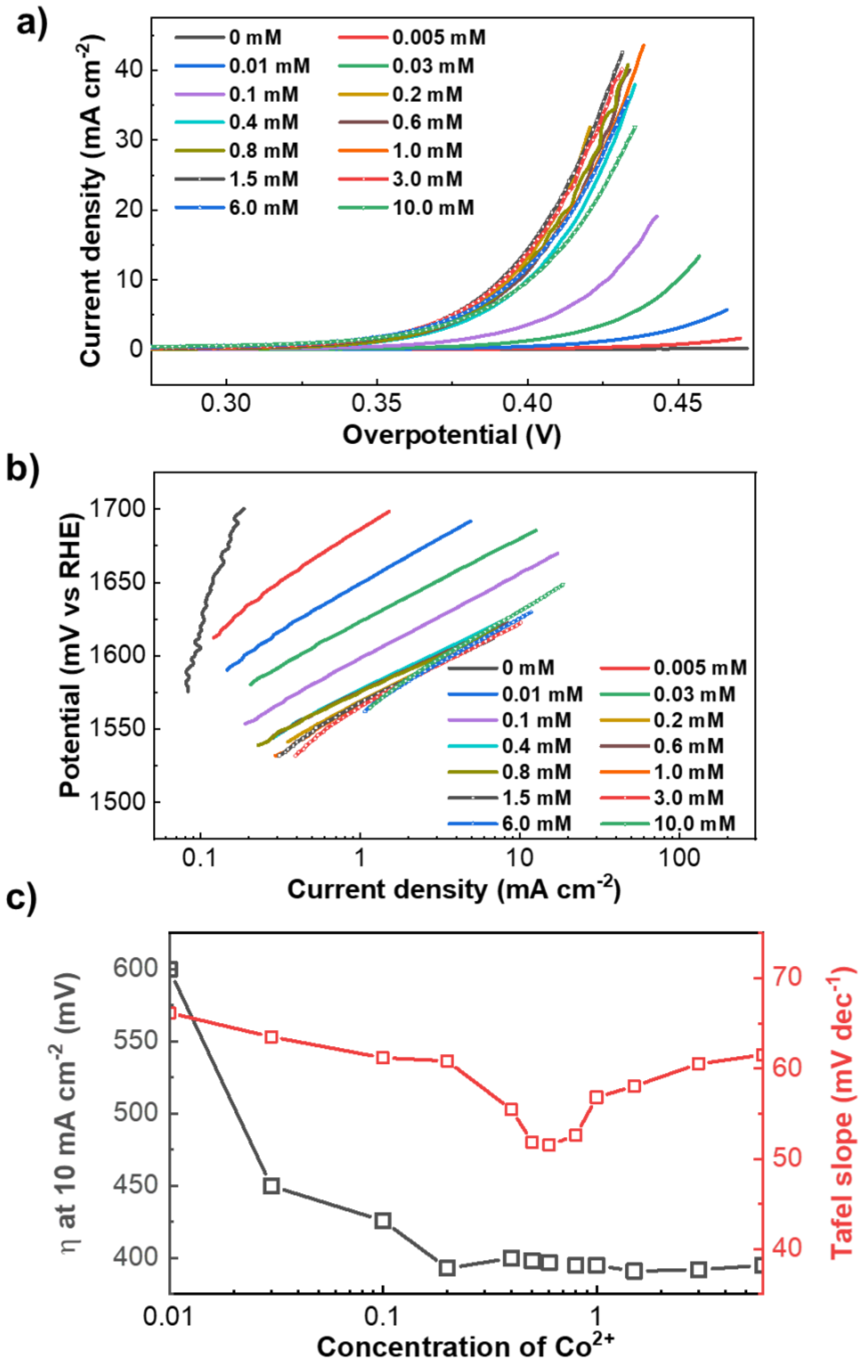

Figure S21. Activity for OER at different concentration of Co^{2+} in the solution a) Current density as a function of overpotential. b) Tafel slope. c) Summary of the overpotential at 10 mA cm^{-2} and the Tafel slope with respect to the concentration of Co^{2+}.

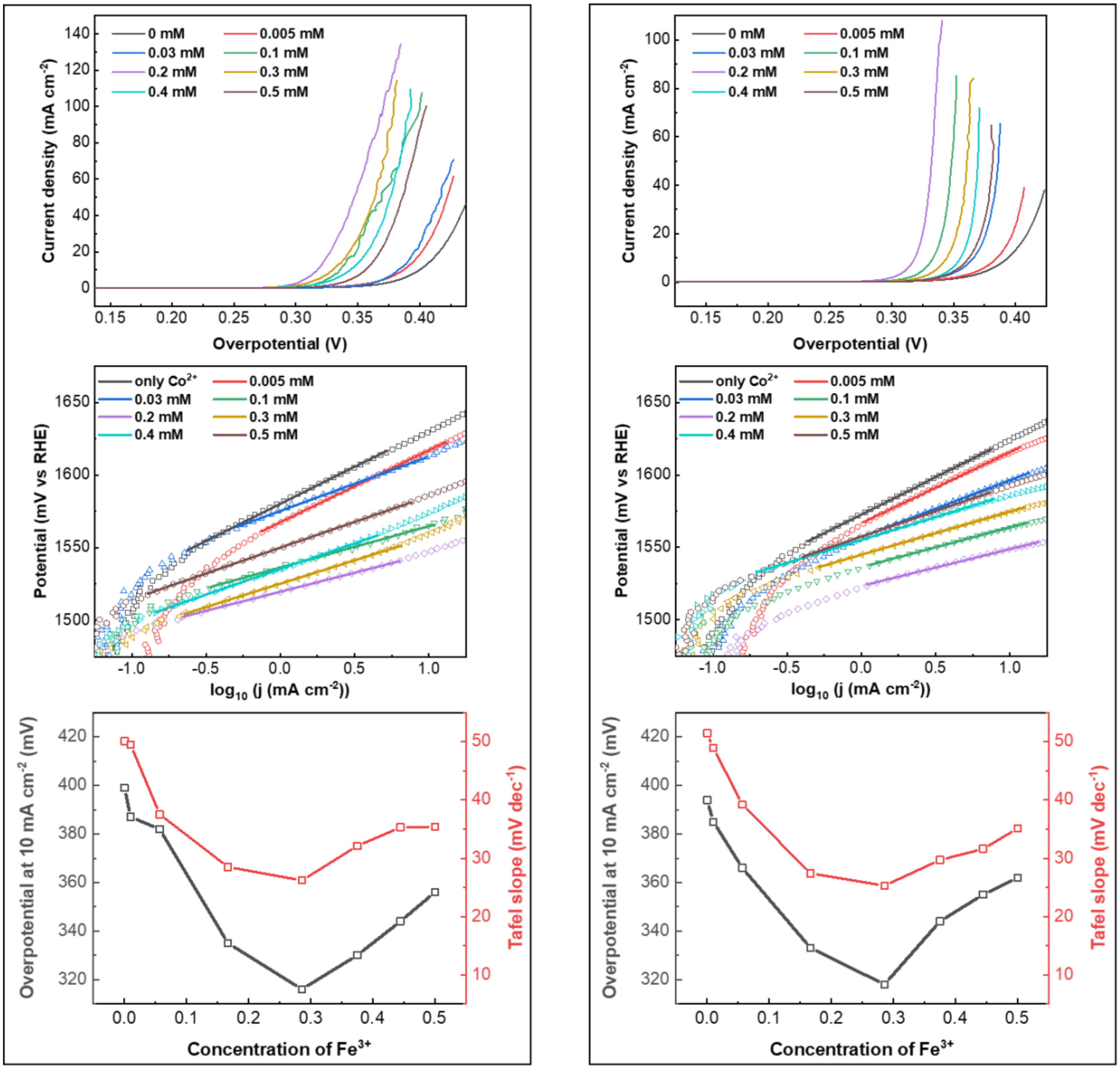

Figure S22. Evolution of the CV scan at 10 mV s^{-1} for various concentration of Fe^{3+} in the electrolyte, with a fixed concentration of Co^{2+} of 0.5 mM. Each sub-figure represents a set of measurements. From top to bottom: current density as a function of overpotential, Tafel slope, overpotential at 10 mA cm^{-2} and Tafel slope over, as a function Fe^{3+} concentration.

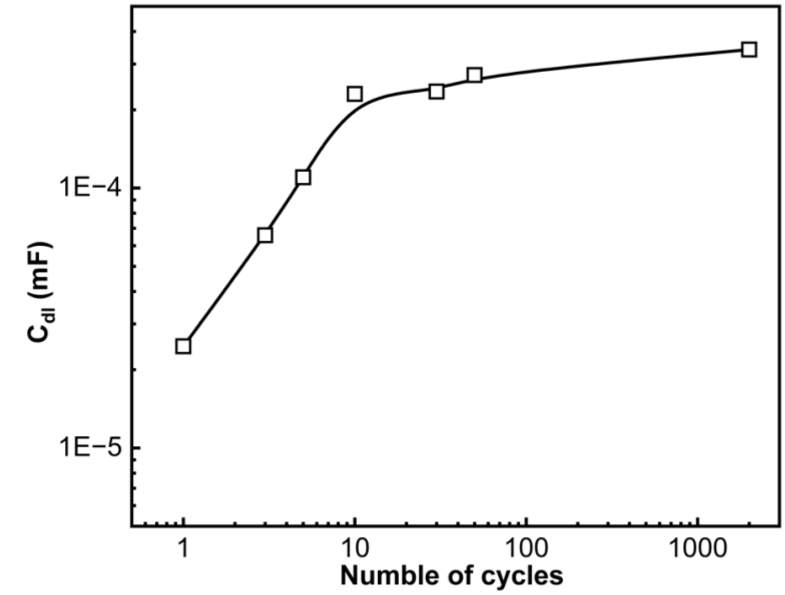

Figure S23. Evolution of the C_{dl} with respect to the number of cycles in KOH-CoFe. The C_{dl} was evaluated after 1, 3, 5, 10, 50, 300 and 2000 cycles.

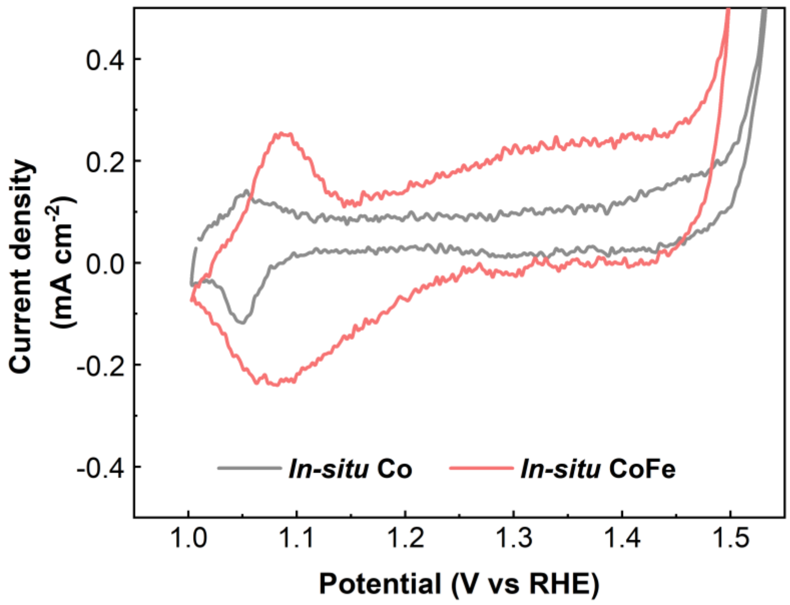

Figure S24. CV curves of in situ Co and in situ Co-Fe in the potential range prior to OER.

Table S5. Summary of the overpotential at 10 mA cm^{-2} and the Tafel slope of 10 different samples, measured after 10 CVs in KOH-Co.

	**Overpotential at 10 mA cm^{-2} (mV)**	**Tafel slope (mV dec^{-1})**
**1**	389	51.4
**2**	394	50.6
**3**	412	56.3
**4**	398	49.1
**5**	401	50.7
**6**	395	52.2
**7**	389.4	49.6
**8**	393	51.7
**9**	392	51.6
**10**	390	51.3
**Average**	395 ± 6.99	51.5 ± 1.96

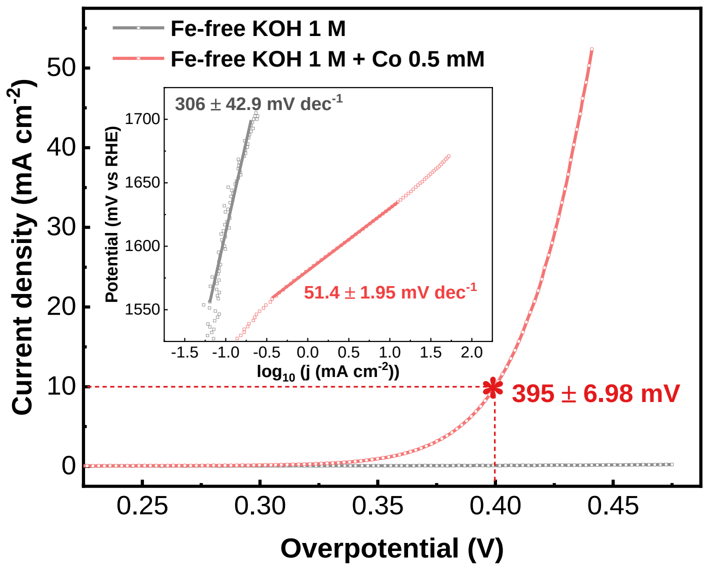

Figure S25. CV curves of GC-RDE in Fe-free KOH and in KOH-Co after 10 cycles. Inset: Tafel slopes. The scan range of the CVs were 1.0 to 1.7 V vs RHE and the scan rate was 10 mV s^{-1}. The CV curves and Tafel slopes were averaged over 10 individual measurements (**Table S5**). All the CV curves were corrected with 85% of iR-drop.

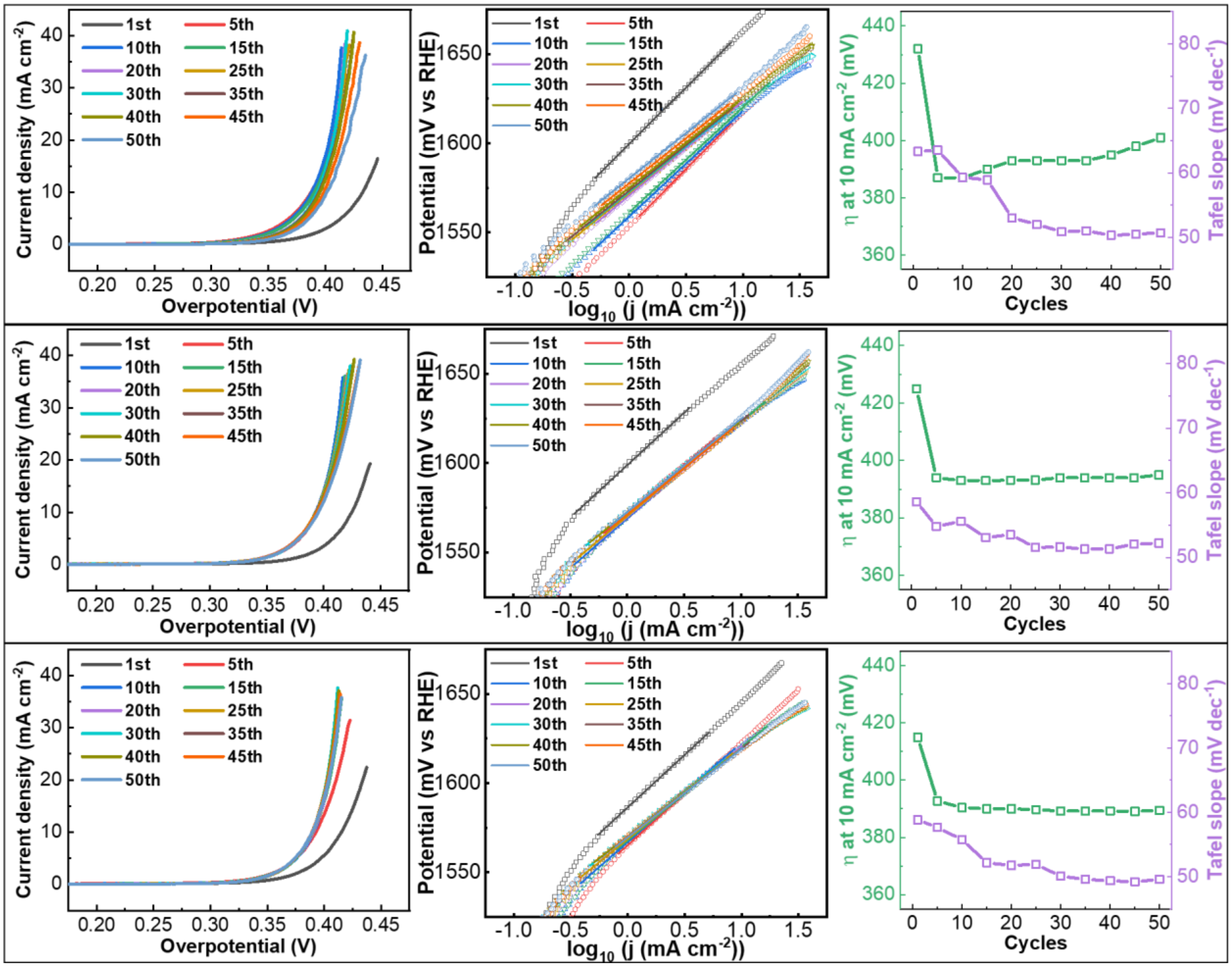

Figure S26. Evolution of the CV scan at 10 mV s^{-1} over 50 cycles in KOH-Co. Each sub-figure represents a set of measurements. From left to right: current density as a function of overpotential, Tafel slope, overpotential at 10 mA cm^{-2} and Tafel slope over as a function of number of cycles.

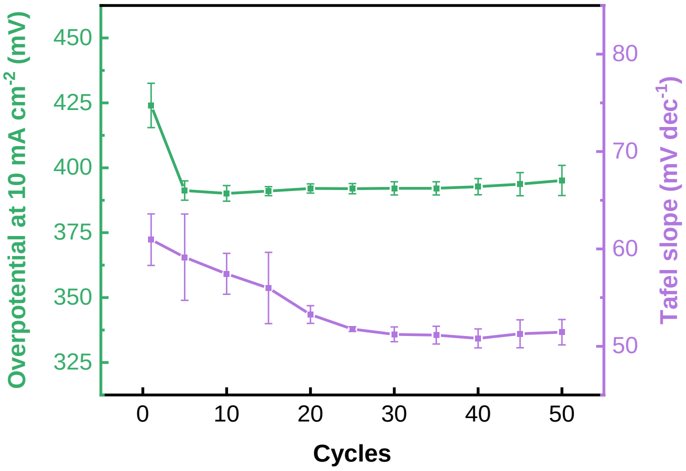

Figure S27. Evolution of the overpotential at 10 mA cm^{-2} and the Tafel slope over 50 CVs in KOH-Co. The scan rate was 10 mV s^{-1}. The error bar was averaged from 3 sets of measurements in **Figure S26**.

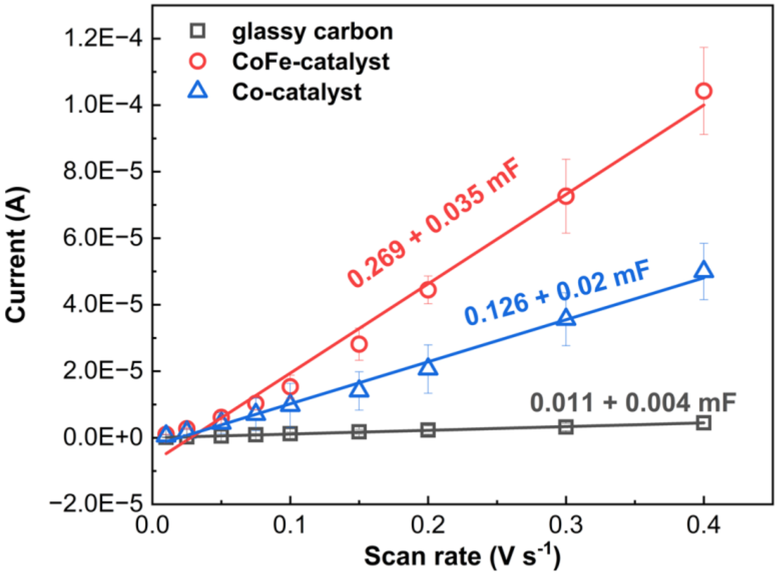

Figure S28. ECSA of the bare GC-RDE, in situ Co and Co-Fe catalysts, acquired after 10 CVs in their corresponding electrolyte for synthesis.

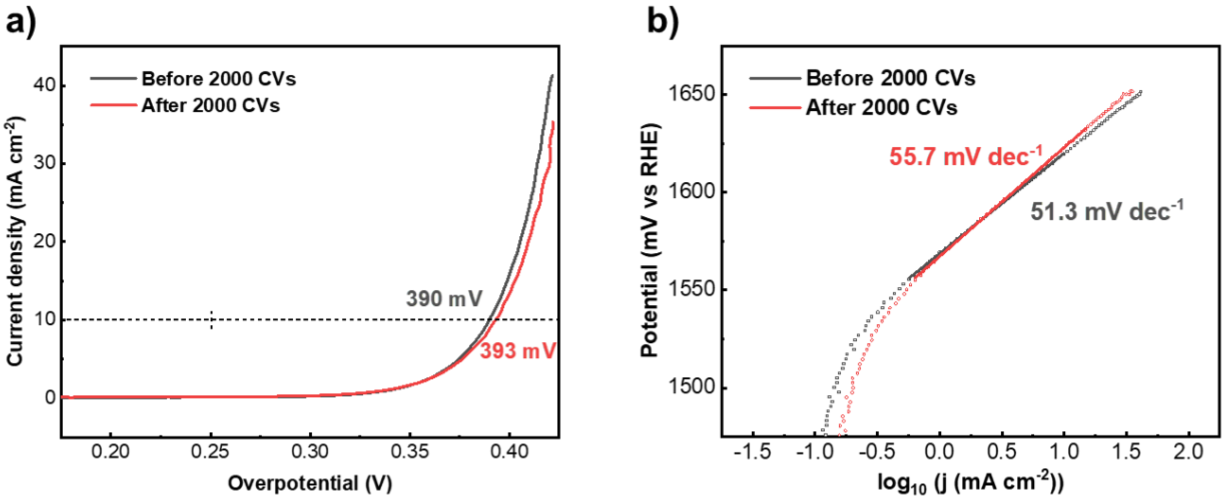

Figure S29. Activity for OER before and after 2000 accelerated CVs in KOH-Co at 400 mV s^{-1} scan rate.

Table S6. Summary of Tafel slope, overpotential at 10 mA cm^{-2} and concentration of KOH from a variety of Co-based catalyst.

Co-based catalysts	Tafel slope (mV dec^{-1})	Overpotential at 10 mA cm^{-2} (mV)	c_{KOH}	References
**In situ Co**	51.4	397	1 M	This work
**ED-CoOOH**	60	430	0.1 M	Ref [1]
**PCP-CoOOH**	51	385	0.1 M	Ref [2]
**Pristine Co_3O_4**	54	398	1 M	Ref [3]
**Co_3O_4/rmGO hybrid**	68	320	1 M	Ref [4]
**Co_3O_4/N-rmGO hybrid**	67	310	1 M	Ref [4]
**Co NPs**	68.2	380	0.1 M	Ref [5]
**CoOOH PNSAs/CFC**	56.4	331	1 M	Ref [6]
**c-Co_3O_4**	53	440	1 M	Ref [7]
**n-Co_3O_4**	153	380	1 M	Ref [7]
**Meso-Co_3O_4**	78	411	1 M	Ref [8]
**Co-Co-N-C**	57	415	1 M	Ref [9]
**Co-Co bulk LDH**	59	393	1 M	Ref [10]
**Co-Co exfoliated LDH**	45	353	1 M	Ref [10]
**Co-N-C precatalyst**	72	495	1 M	Ref [11]

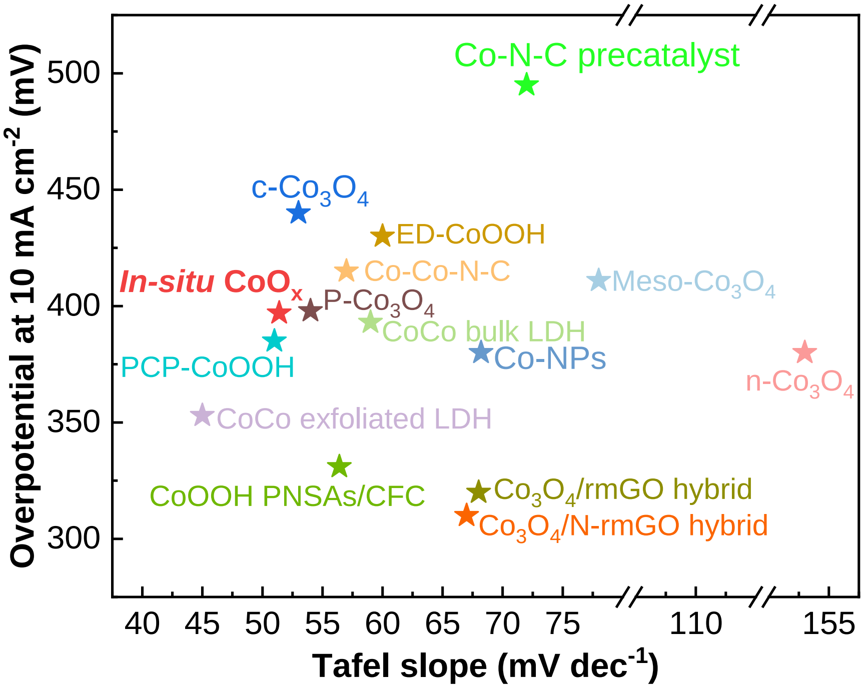

Figure S30. OER activity of the in situ Co catalyst compared with other reported Co-based catalyst. The details of the catalysts and electrolyte are summarized in **Table S6**.

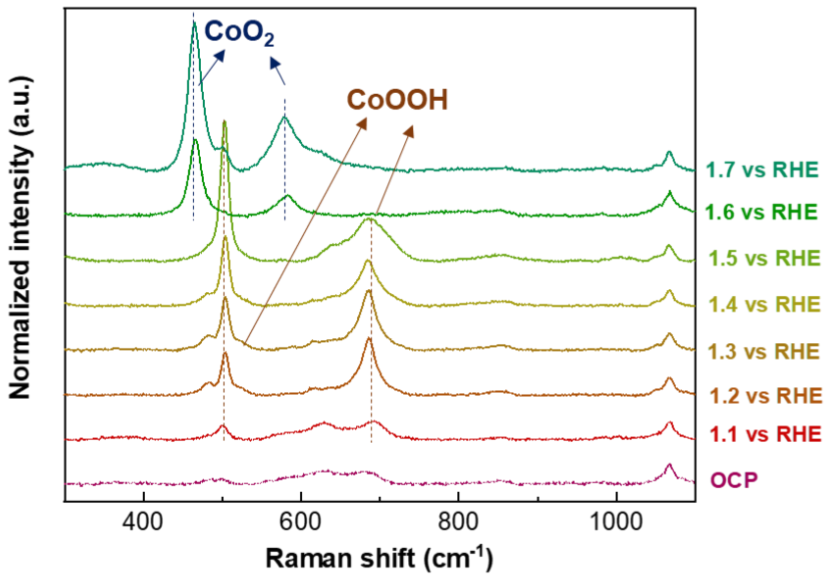

Figure S31. Operando Raman measurements acquired on GC plate in KOH-Co.

Table S7. Summary of the reported Raman peak positions for CoOOH and OER-active phase CoO_2.

References	CoOOH (cm^{-1})	CoO_2 (cm^{-1})
Mechanism of Oxygen Evolution Catalyzed by Cobalt Oxyhydroxide: Cobalt Superoxide Species as a Key Intermediate and Dioxygen Release as a Rate-Determining Step[1]	500	474 (E_g), 581 (A_{1g})
Electrochemical Construction of Low-Crystalline CoOOH Nanosheets with Short-Range Ordered Grains to Improve Oxygen Evolution Activity[12]	502, 684	
Reversible Structural Evolution of $NiCoO_xH_y$ during the Oxygen Evolution Reaction and Identification of the Catalytically Active Phase[13]		467 (E_g), 572 (A_{1g})
Understanding the Roles of Electrogenerated Co^{3+} and Co^{4+} in Selectivity-Tuned 5-Hydroxymethylfurfural Oxidation[14]	503 (E_g)	474 (E_g), 560 (A_{1g})
Raman Study of Layered Rock-Salt $LiCoO_2$ and Its Electrochemical Lithium Deintercalation[15]		460 (E_g), 570 (A_{1g}) ($Li_{0.4}CoO_2$)
Enhanced Activity of Gold-Supported Cobalt Oxide for the Electrochemical Evolution of Oxygen[16]	505, 565	

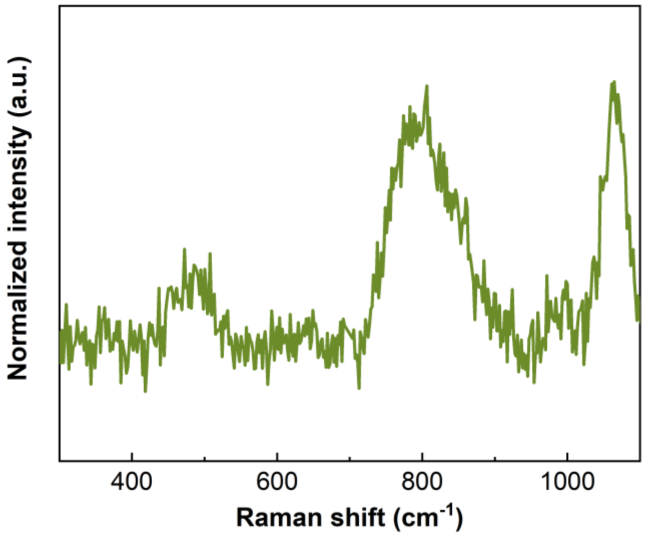

Figure S32. Raman spectrum of bare GC plate surface.

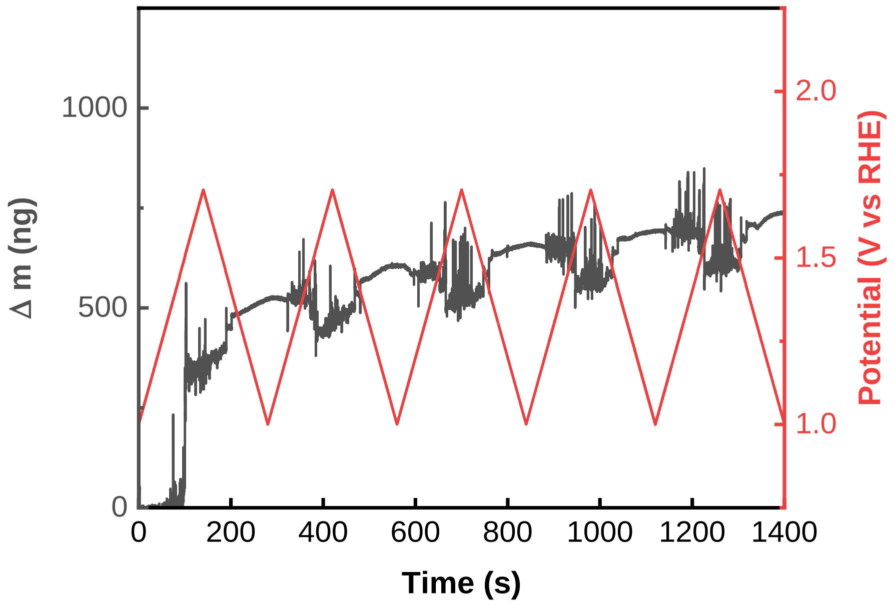

Figure S33. Change in mass of in situ Co catalyst over 5 CVs. The drop of mass occurred when the potential went up from 1.55 to 1.7 then down to 1.55 vs RHE.

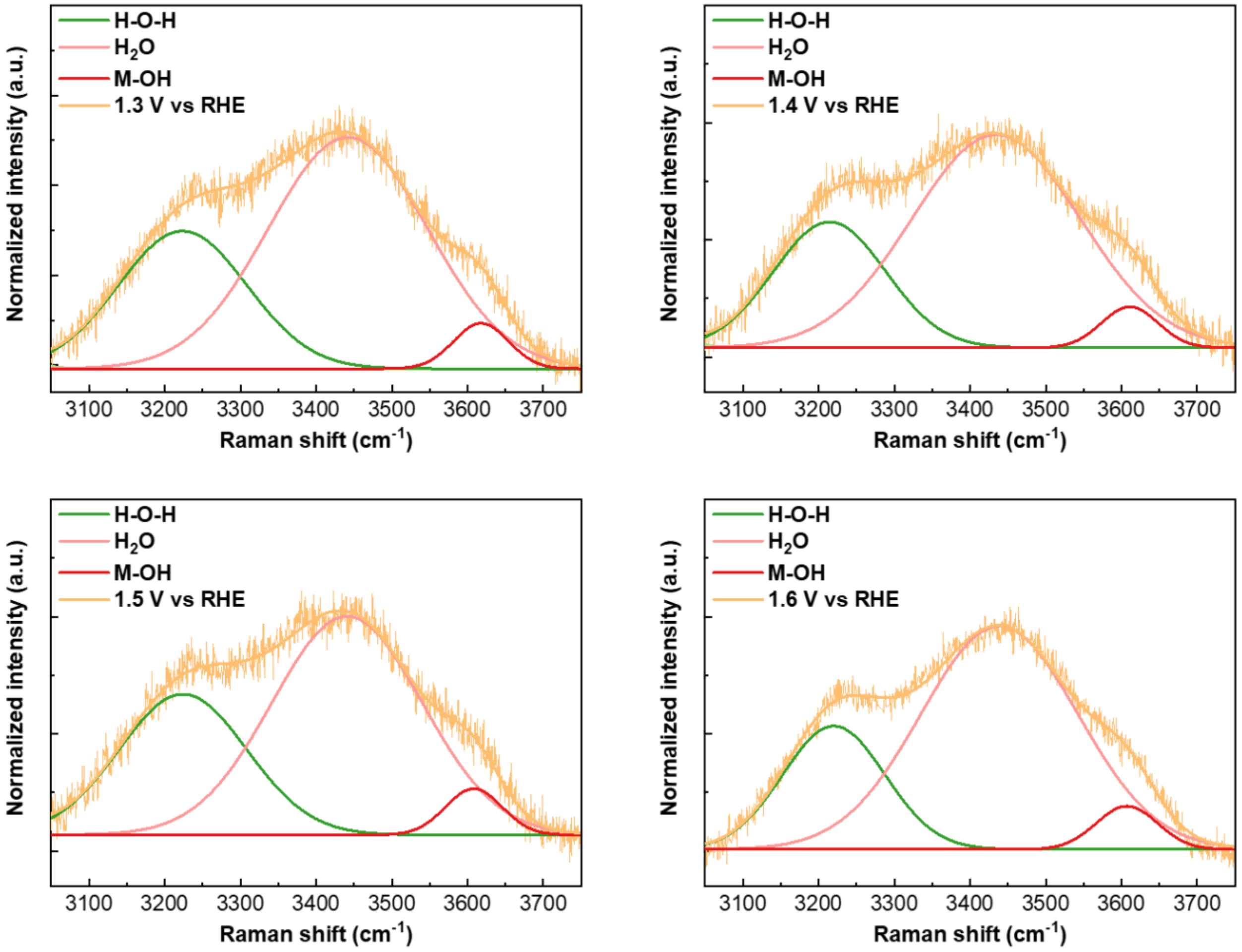

Figure S34. Operando Raman spectroscopy and the deconvoluted peaks of the GC plate activated in KOH-Co in the range of 3050 to 3750 cm^{-1}. The applied potentials were from 1.3 to 1.6 V vs RHE.

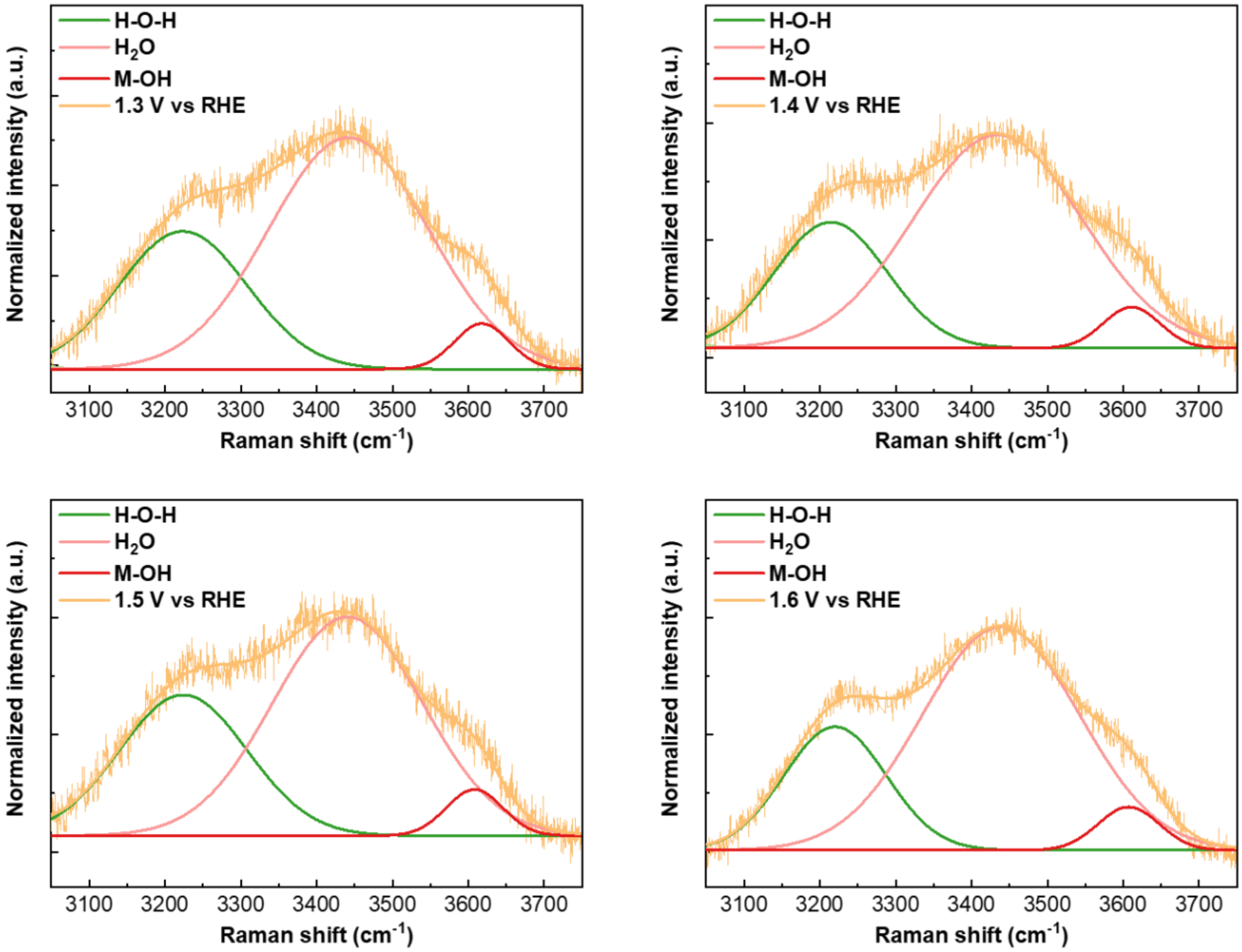

Figure S35. Operando Raman spectroscopy and the deconvoluted peaks of the GC plate activated in KOH-CoFe in the range of 3050 to 3750 cm^{-1}. The applied potentials were from 1.3 to 1.6 V vs RHE.

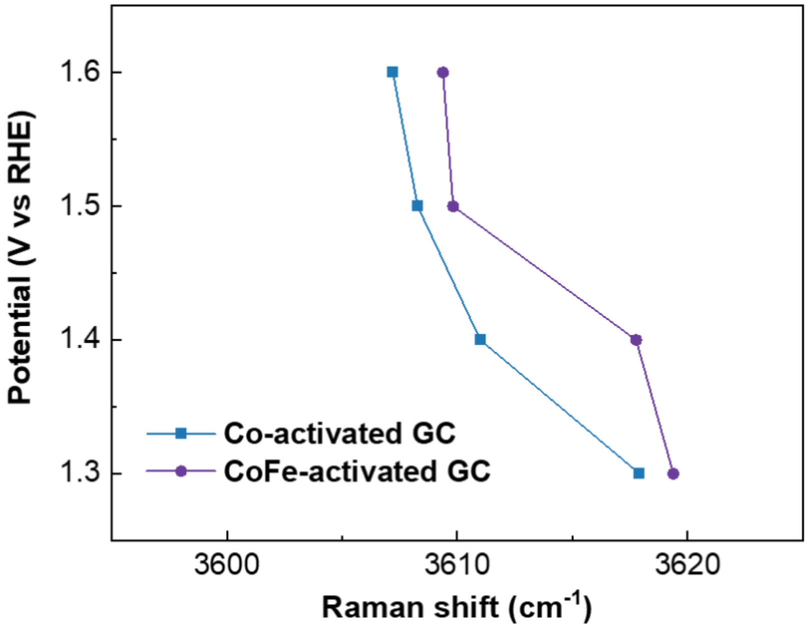

Figure S36. Position of the M-OH bond peak at different applied potentials for GC plate activated in KOH-Co and in KOH-CoFe.

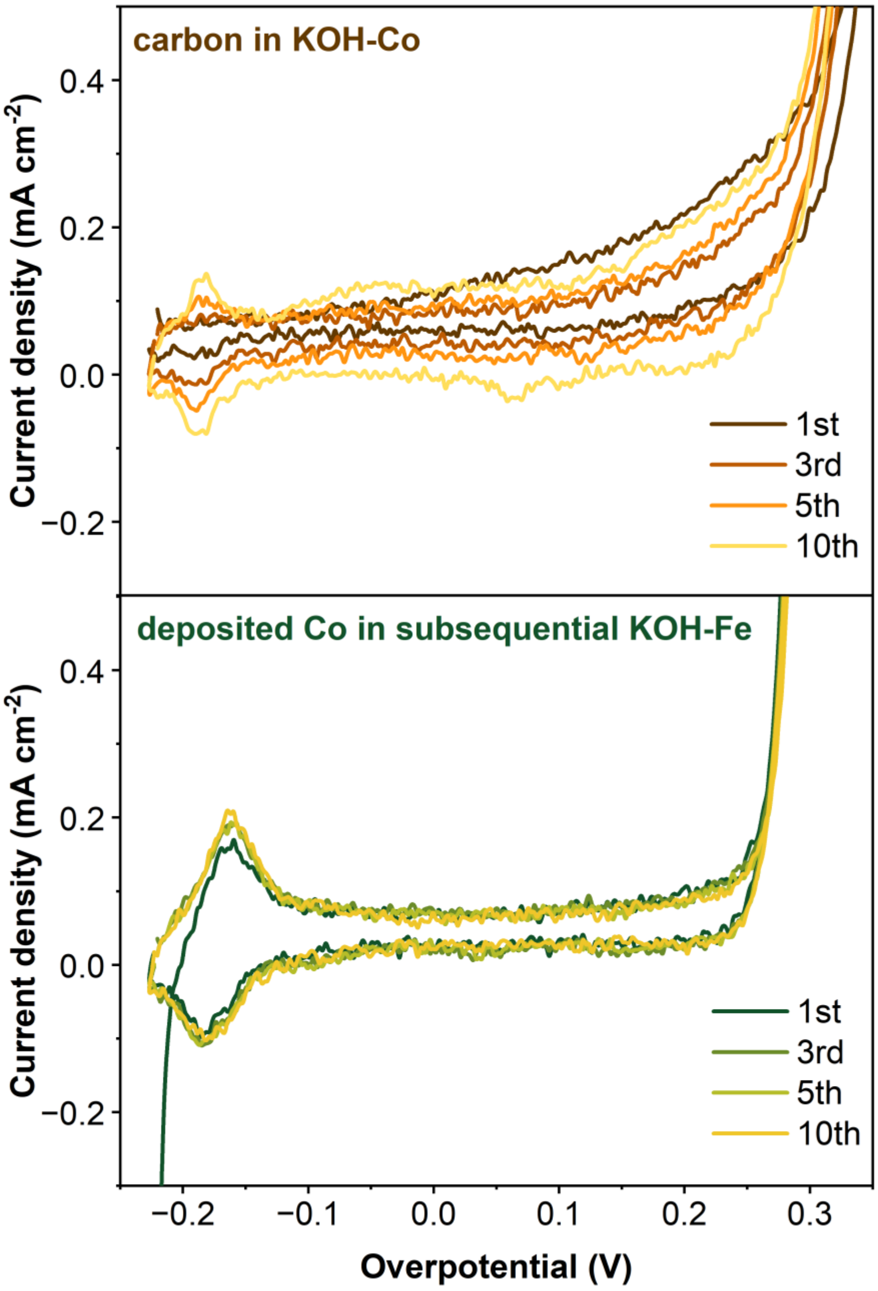

Figure S37. CV curve of GC-RDE cycled in KOH-Co, and that of deposited Co cycled in KOH-Fe, in the potential range prior to OER. The 1st, 3rd, 5th and 10th CV curves were presented to follow to evolution of electrochemical profile upon cycling.

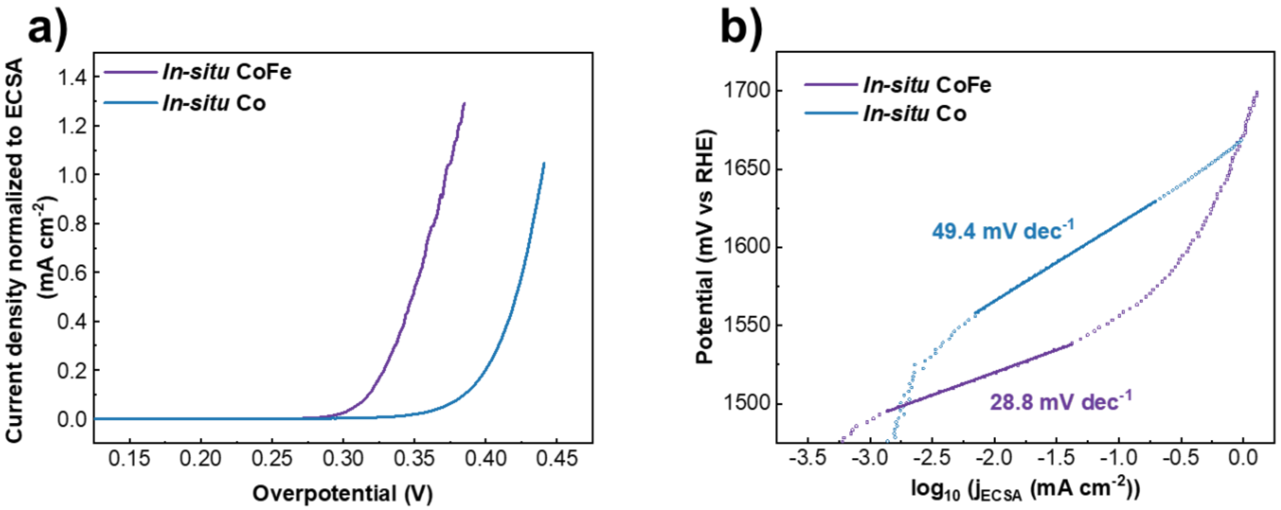

Figure S38. a) ECSA-normalized current density as a function of overpotential of in situ Co and Co-Fe catalysts. b) and the corresponding Tafel slopes.

Table S8. Summary of Tafel slope, overpotential at 10 mA cm^{-2} and concentration of KOH from a variety of Co-Fe-based catalyst, IrO_2 and RuO_2.

Co-Fe-based catalyst	Tafel slope (mV dec^{-1})	Overpotential at 10 mA cm^{-2} (mV)	c_{KOH}	Ref
**In situ Co-Fe**	28.6	320	1 M	This work
**$Fe_{0.33}Co_{0.67}OOH$ PNSAs/CFC**	30	266	1 M	Ref [6]
**Co/Fe 32 red**	41	339	1 M	Ref [17]
**Co/Fe 32**	55	378	1M	Ref [3]
**$Co_{0.46}Fe_{0.54}OOH$**	29	330	1 M	Ref [18]
**Co_3Fe_7**	70.8	440	0.1 M	Ref [5]
**Co disk activated in Fe^{3+}**	33	345	1 M	Ref [19]
**Fe adsorbed CoO_x**	27.6	309	1 M	Ref [20]
**Co-Fe-N-C on carbon cloth**	40	321	1 M	Ref [9]
**Co-Fe-N-C on glassy carbon**	44	360	1 M	Ref [11]
**Co(Fe)OOH**	55	300	0.1 M	Ref [2]
**Fe-CoOOH/G**	37	330	1 M	Ref [21]
**$FeCo_2O_4$**	83.2	470	0.1 M	Ref [22]
**Co(Fe)OOH_x nanoparticles (2.8%Fe)**	43	556	1 M	Ref [23]
**Fe_1Co_3Ox@C-800**	40	272	1 M	Ref [24]
**$Co_xFe_{1-x}P$/NC**	44	299	1 M	Ref [25]
**CoFe-7.5**	58	294	1 M	Ref [8]
**BSCF**	60	360	0.1 M	Ref [26]
**$CoFe_2O_4$**	82.15	403	0.1 M	Ref [27]
**RuO_2 || C**	52.7	328	1M	This work
**IrO_2 || C**	72.8	491	1M	This work

REFERENCES

(1) Moysiadou, A.; Lee, S.; Hsu, C. S.; Chen, H. M.; Hu, X. Mechanism of Oxygen Evolution Catalyzed by Cobalt Oxyhydroxide: Cobalt Superoxide Species as a Key Intermediate and Dioxygen Release as a Rate-Determining Step. *J. Am. Chem. Soc.* **2020**, *142* (27), 11901–11914. https://doi.org/10.1021/jacs.0c04867.

(2) Cheng, X.; Kim, B. J.; Fabbri, E.; Schmidt, T. J. Co/Fe Oxyhydroxides Supported on Perovskite Oxides as Oxygen Evolution Reaction Catalyst Systems. *ACS Appl. Mater. Interfaces* **2019**, *11* (38), 34787–34795. https://doi.org/10.1021/acsami.9b04456.

(3) Budiyanto, E.; Yu, M.; Chen, M.; Debeer, S.; Rüdiger, O.; Tüysüz, H. Tailoring Morphology and Electronic Structure of Cobalt Iron Oxide Nanowires for Electrochemical Oxygen Evolution Reaction. *ACS Appl. Energy Mater.* **2020**, *3* (9), 8583–8594. https://doi.org/10.1021/acsaem.0c01201.

(4) Liang, Y.; Li, Y.; Wang, H.; Zhou, J.; Wang, J.; Regier, T.; Dai, H. Co3O4 Nanocrystals on Graphene as a Synergistic Catalyst for Oxygen Reduction Reaction. *Nat. Mater.* **2011**, *10*, 780–786. https://doi.org/10.1038/nmat3087.

(5) Meng, J.; Cui, Z.; Yang, X.; Zhu, S.; Li, Z.; Qi, K.; Zheng, L.; Liang, Y. Cobalt-Iron (Oxides) Water Oxidation Catalysts: Tracking Catalyst Redox States and Reaction Dynamic Mechanism. *J. Catal.* **2018**, *365*, 227–237. https://doi.org/10.1016/J.JCAT.2018.06.031.

(6) Ye, S. H.; Shi, Z. X.; Feng, J. X.; Tong, Y. X.; Li, G. R. Activating CoOOH Porous Nanosheet Arrays by Partial Iron Substitution for Efficient Oxygen Evolution Reaction. *Angew. Chemie - Int. Ed.* **2018**, *57* (10), 2672–2676. https://doi.org/10.1002/anie.201712549.

(7) Alex, C.; Sarma, S. C.; Peter, S. C.; John, N. S. Competing Effect of Co3+Reducibility and Oxygen-Deficient Defects Toward High Oxygen Evolution Activity in Co3O4Systems in Alkaline Medium. *ACS Appl. Energy Mater.* **2020**, *3* (6), 5439–5447. https://doi.org/10.1021/acsaem.0c00297.

(8) Gao, X.; Liu, J.; Sun, Y.; Wang, X.; Geng, Z.; Shi, F.; Wang, X.; Zhang, W.; Feng, S.; Wang, Y.; Huang, K. Optimized Co2+(Td)-O-Fe3+(Oh) Electronic States in a Spinel Electrocatalyst for Highly Efficient Oxygen Evolution Reaction Performance. *Inorg. Chem. Front.* **2019**, *6* (11), 3295–3301. https://doi.org/10.1039/c9qi00852g.

(9) Bai, L.; Hsu, C. S.; Alexander, D. T. L.; Chen, H. M.; Hu, X. Double-Atom Catalysts as a Molecular Platform for Heterogeneous Oxygen Evolution Electrocatalysis. *Nat. Energy* **2021**, *6* (11), 1054–1066. https://doi.org/10.1038/s41560-021-00925-3.

(10) Song, F.; Hu, X. Exfoliation of Layered Double Hydroxides for Enhanced Oxygen Evolution Catalysis. *Nat. Commun.* **2014**, *5*, 4477. https://doi.org/10.1038/ncomms5477.

(11) Bai, L.; Hsu, C.-S.; Alexander, D. T. L.; Chen, H. M.; Hu, X. A Cobalt–Iron Double-Atom Catalyst for the Oxygen Evolution Reaction. *J. Am. Chem. Soc.* **2019**, *141* (36), 14190–14199. https://doi.org/10.1021/JACS.9B05268.

(12) Ye, S.; Wang, J.; Hu, J.; Chen, Z.; Zheng, L.; Fu, Y.; Lei, Y.; Ren, X.; He, C.; Zhang, Q.; Liu, J. Electrochemical Construction of Low-Crystalline CoOOH Nanosheets with Short-Range Ordered Grains to Improve Oxygen Evolution Activity. *ACS Catalysis*. **2021**, *11 (10)*, 6104–6112. https://doi.org/10.1021/acscatal.1c01300.

(13) Chen, Z.; Cai, L.; Yang, X.; Kronawitter, C.; Guo, L.; Shen, S.; Koel, B. E. Reversible Structural

Evolution of NiCoOxHy during the Oxygen Evolution Reaction and Identification of the Catalytically Active Phase. *ACS Catal.* **2018**, *8* (2), 1238–1247. https://doi.org/10.1021/acscatal.7b03191.

(14) Deng, X.; Xu, G. Y.; Zhang, Y. J.; Wang, L.; Zhang, J.; Li, J. F.; Fu, X. Z.; Luo, J. L. Understanding the Roles of Electrogenerated Co3+ and Co4+ in Selectivity-Tuned 5-Hydroxymethylfurfural Oxidation. *Angew. Chemie - Int. Ed.* **2021**, *60* (37), 20535–20542. https://doi.org/10.1002/anie.202108955.

(15) Inaba, M.; Iriyama, Y.; Ogumi, Z.; Todzuka, Y.; Tasaka, A. Raman Study of Layered Rock-Salt LiCoO2 and Its Electrochemical Lithium Deintercalation. *J. Raman Spectrosc.* **1997**, *28 (8)*, 613–617. https://doi.org/10.1002/(SICI)1097-4555(199708)28:8<613::AID-JRS138>3.0.CO;2-T.

(16) Yeo, B. S.; Bell, A. T. Enhanced Activity of Gold-Supported Cobalt Oxide for the Electrochemical Evolution of Oxygen. *J. Am. Chem. Soc.* **2011**, *133* (14), 5587–5593. https://doi.org/10.1021/ja200559j.

(17) Budiyanto, E.; Salamon, S.; Wang, Y.; Wende, H.; Tü Ysü, H. Phase Segregation in Cobalt Iron Oxide Nanowires toward Enhanced Oxygen Evolution Reaction Activity. *JACS Au* **2022**, *2* (3), 697–710. https://doi.org/10.1021/JACSAU.1C00561.

(18) Burke, M. S.; Kast, M. G.; Trotochaud, L.; Smith, A. M.; Boettcher, S. W. Cobalt-Iron (Oxy)Hydroxide Oxygen Evolution Electrocatalysts: The Role of Structure and Composition on Activity, Stability, and Mechanism. *J. Am. Chem. Soc.* **2015**, *137* (10), 3638–3648. https://doi.org/10.1021/jacs.5b00281.

(19) Gong, L.; Koh, J.; Yeo, B. S. Mechanistic Study of the Synergy between Iron and Transition Metals for the Catalysis of the Oxygen Evolution Reaction. *ChemSusChem* **2018**, *11* (21), 3790–3795. https://doi.org/10.1002/CSSC.201801639.

(20) Gong, L.; Chng, X. Y. E.; Du, Y.; Xi, S.; Yeo, B. S. Enhanced Catalysis of the Electrochemical Oxygen Evolution Reaction by Iron(III) Ions Adsorbed on Amorphous Cobalt Oxide. *ACS Catal.* **2017**, *8* (2), 807–814. https://doi.org/10.1021/ACSCATAL.7B03509.

(21) Han, X.; Yu, C.; Zhou, S.; Zhao, C.; Huang, H.; Yang, J.; Liu, Z.; Zhao, J.; Qiu, J. Ultrasensitive Iron-Triggered Nanosized Fe–CoOOH Integrated with Graphene for Highly Efficient Oxygen Evolution. *Adv. Energy Mater.* **2017**, *7* (14), 1602148. https://doi.org/10.1002/aenm.201602148.

(22) Harada, M.; Kotegawa, F.; Kuwa, M. Structural Changes of Spinel MCo2O4(M = Mn, Fe, Co, Ni, and Zn) Electrocatalysts during the Oxygen Evolution Reaction Investigated by in Situ X-Ray Absorption Spectroscopy. *ACS Appl. Energy Mater.* **2022**, *5* (1), 278–294. https://doi.org/10.1021/acsaem.1c02824.

(23) Sun, Z.; Curto, A.; Rodríguez-Fernández, J.; Wang, Z.; Parikh, A.; Fester, J.; Dong, M.; Vojvodic, A.; Lauritsen, J. V. The Effect of Fe Dopant Location in Co(Fe)OOHxNanoparticles for the Oxygen Evolution Reaction. *ACS Nano* **2021**, *15* (11), 18226–18236. https://doi.org/10.1021/acsnano.1c07219.

(24) Bai, X.; Wang, Q.; Guan, J. Bimetallic Iron-Cobalt Nanoparticles Coated with Amorphous Carbon for Oxygen Evolution. *ACS Appl. Nano Mater.* **2021**, *4* (11), 12663–12671. https://doi.org/10.1021/acsanm.1c03208.

(25) Chen, J.; Zhang, Y.; Ye, H.; Xie, J. Q.; Li, Y.; Yan, C.; Sun, R.; Wong, C. P. Metal-Organic

Framework-Derived CoxFe1- XP Nanoparticles Encapsulated in N-Doped Carbon as Efficient Bifunctional Electrocatalysts for Overall Water Splitting. *ACS Appl. Energy Mater.* **2019**, *2* (4), 2734–2742. https://doi.org/10.1021/acsaem.9b00065.

(26) Suntivich, J.; May, K. J.; Gasteiger, H. A.; Goodenough, J. B.; Shao-Horn, Y. A Perovskite Oxide Optimized for Oxygen Evolution Catalysis from Molecular Orbital Principles. *Science (80-.).* **2011**, *334* (6061), 1383–1385. https://doi.org/10.1126/science.1212858.

(27) Li, M.; Xiong, Y.; Liu, X.; Bo, X.; Zhang, Y.; Han, C.; Guo, L. Facile Synthesis of Electrospun MFe2O4 (M = Co, Ni, Cu, Mn) Spinel Nanofibers with Excellent Electrocatalytic Properties for Oxygen Evolution and Hydrogen Peroxide Reduction. *Nanoscale* **2015**, *7* (19), 8920–8930. https://doi.org/10.1039/C4NR07243J.
